# Myocardial fibrosis and calcification are attenuated by microRNA–129-5p targeting Asporin and Sox9 in cardiac fibroblasts

**DOI:** 10.1172/jci.insight.168655

**Published:** 2023-05-08

**Authors:** Lejla Medzikovic, Laila Aryan, Grégoire Ruffenach, Min Li, Nicoletta Savalli, Wasila Sun, Shervin Sarji, Jason Hong, Salil Sharma, Riccardo Olcese, Gregory Fishbein, Mansoureh Eghbali

**Affiliations:** 1 Department of Anesthesiology & Perioperative Medicine,; 2 Division of Pulmonary & Critical Care Medicine,; 3 Department of Physiology, and; 4 Department of Pathology & Laboratory Medicine, David Geffen School of Medicine at UCLA, Los Angeles, California, USA.

**Keywords:** Cardiology, Fibrosis, Heart failure, Molecular biology

## Abstract

Myocardial fibrosis and calcification associate with adverse outcomes in nonischemic heart failure. Cardiac fibroblasts (CF) transition into myofibroblasts (MF) and osteogenic fibroblasts (OF) to promote myocardial fibrosis and calcification. However, common upstream mechanisms regulating both CF-to-MF transition and CF-to-OF transition remain unknown. microRNAs are promising targets to modulate CF plasticity. Our bioinformatics revealed downregulation of miR–129-5p and upregulation of its targets small leucine–rich proteoglycan Asporin (ASPN) and transcription factor SOX9 as common in mouse and human heart failure (HF). We experimentally confirmed decreased miR–129-5p and enhanced SOX9 and ASPN expression in CF in human hearts with myocardial fibrosis and calcification. miR–129-5p repressed both CF-to-MF and CF-to-OF transition in primary CF, as did knockdown of SOX9 and ASPN. *Sox9* and *Aspn* are direct targets of miR–129-5p that inhibit downstream β-catenin expression. Chronic Angiotensin II infusion downregulated miR–129-5p in CF in WT and TCF21-lineage CF reporter mice, and it was restored by miR–129-5p mimic. Importantly, miR–129-5p mimic not only attenuated progression of myocardial fibrosis, calcification marker expression, and SOX9 and ASPN expression in CF but also restored diastolic and systolic function. Together, we demonstrate miR–129-5p/ASPN and miR–129-5p/SOX9 as potentially novel dysregulated axes in CF-to-MF and CF-to-OF transition in myocardial fibrosis and calcification and the therapeutic relevance of miR–129-5p.

## Graphical abstract

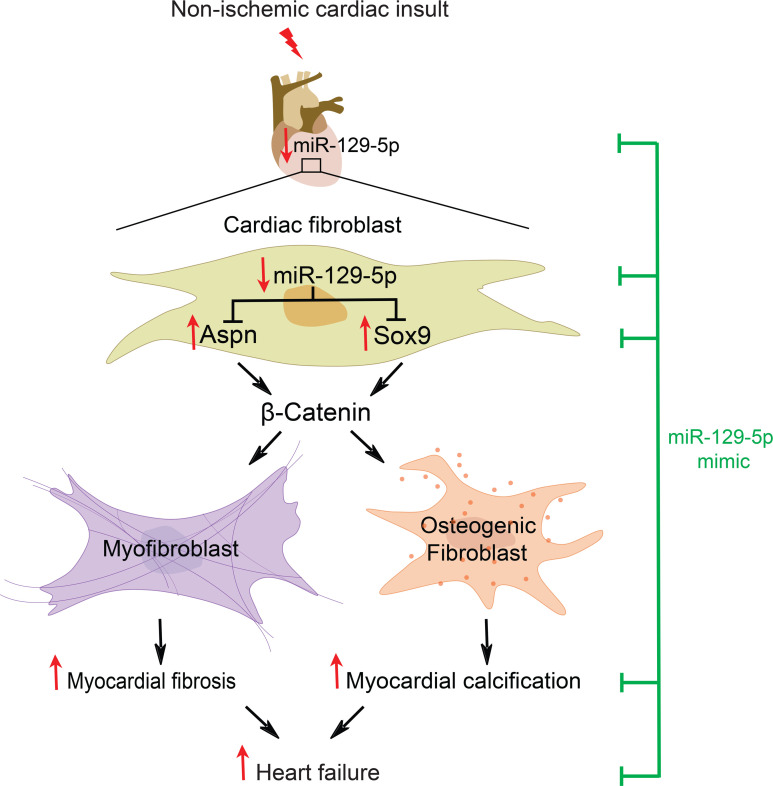

## Introduction

Nonischemic heart failure (HF) remains a leading cause of death, and cardiac fibrosis critically contributes to HF progression by impairing myocardial compliance, contractility, and conductance (1). Unsurprisingly, cardiac fibrosis is an independent mortality predictor in patients with nonischemic HF (2, 3). Besides excessive extracellular matrix synthesis, another pathological aspect of cardiac fibrosis is predisposition for myocardial calcification. Myocardial calcification has been reported (4) to occur in sepsis (5, 6), renal disease and failure (5–8), and areas of endomyocardial fibrosis (9, 10). Indeed, myocardial calcification is one of the common causes of cardiac conduction blocks, arrhythmias, and contractile dysfunction and is associated with adverse cardiac outcomes (4, 11, 12).

Resident cardiac fibroblasts (CF) may hold the key to fibrosis and calcification-specific HF therapies. CF activation, transition to profibrotic myofibroblasts (MF), and reversion to a quiescent state all modulate the onset and progression of the fibrotic response (13, 14). Recently, CF have been shown to causally contribute to myocardial calcification by adopting osteogenic cell fates and become osteogenic fibroblasts (OF) after ischemic cardiac injury ,which could be pharmacologically targeted (11). Since myocardial calcification is often observed in areas of fibrosis, a common upstream mechanism that regulates both CF-to-MF transition and CF-to-OF transition may exist but remains to be discovered. This CF plasticity is an attractive therapeutic target, as HF, myocardial fibrosis, and myocardial calcification are often not diagnosed early.

One promising molecular mechanism to target CF plasticity is the small noncoding microRNA (miR). While miRs are known key regulators of cardiac fibrosis and of calcification in the cardiovascular system (15, 16), little is known about the role of miRs that regulate both the fibrotic and osteogenic phenotype in CF. miRs recognize seed regions on mRNAs to inhibit the expression of their target genes by affecting mRNA translation or transcript stability (17). One miR can regulate several different genes, and miRs can be easily manipulated (17). These characteristics provide an attractive opportunity to explore the therapeutic potential of miRs against the progression of myocardial fibrosis and calcification.

Using a bioinformatics and experimental approach in mice and humans, we here show for the potentially novel protective role of miR–129-5p in CF in nonischemic HF, myocardial fibrosis, and calcification. Although the role of miR–129-5p in cardiomyocyte hypertrophy was shown recently (18, 19), our work is the first to our knowledge to investigate the role of miR–129-5p in regulating fibrosis and calcification in the heart by targeting CF plasticity, which is therapeutically more attractive than terminally differentiated cardiomyocytes. We furthermore identify the role of miR–129-5p targets small leucine–rich proteoglycan asporin (ASPN) and transcription factor SOX9 in regulating both CF-to-MF and CF-to-OF transition. Moreover, we demonstrate the therapeutic potential of miR–129-5p in vivo. Our study demonstrates a common mechanism behind MF and OF differentiation in myocardial fibrosis and calcification in nonischemic HF.

## Results

### Bioinformatic analysis reveals downregulated LV expression of miR–129-5p and upregulation of its targets Sox9 and Aspn in mouse and human nonischemic HF.

**Figure 1:**
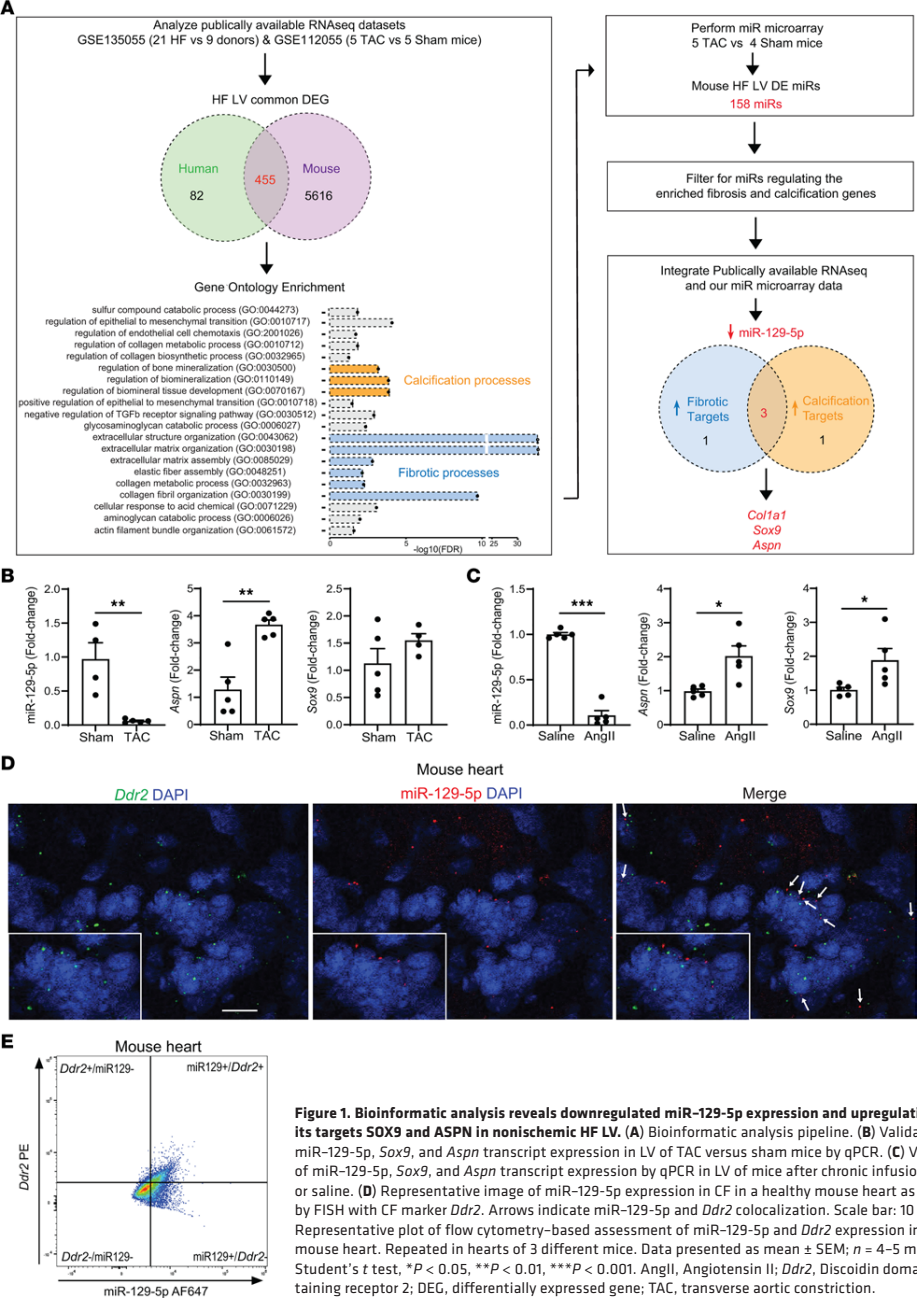
Bioinformatic analysis reveals downregulated miR–129-5p expression and upregulation of its targets SOX9 and ASPN in nonischemic HF LV. (**A**) Bioinformatic analysis pipeline. (**B**) Validation of miR–129-5p, *Sox9*, and *Aspn* transcript expression in LV of TAC versus sham mice by qPCR. (**C**) Validation of miR–129-5p, *Sox9*, and *Aspn* transcript expression by qPCR in LV of mice after chronic infusion of AngII or saline. (**D**) Representative image of miR–129-5p expression in CF in a healthy mouse heart as assessed by FISH with CF marker *Ddr2*. Arrows indicate miR–129-5p and *Ddr2* colocalization. Scale bar: 10 μm. (**E**) Representative plot of flow cytometry–based assessment of miR–129-5p and *Ddr2* expression in a healthy mouse heart. Repeated in hearts of 3 different mice. Data presented as mean ± SEM; *n* = 4–5 mice/group. Student’s *t* test, *P < 0.05, **P < 0.01, ***P < 0.001. AngII, Angiotensin II; *Ddr2*, Discoidin domain-containing receptor 2; DEG, differentially expressed gene; TAC, transverse aortic constriction.

To find novel miRs and targets regulating myocardial fibrosis and calcification in the context of nonischemic HF, we employed an exploratory bioinformatics approach. We compared a publicly available RNA-Seq data set of left ventricles (LV) from patients with nonischemic HF and healthy donors (20) with an RNA-Seq data set of the transverse aortic constriction (TAC) nonischemic HF model in mice (21). In both studies, myocardial fibrosis was apparent in failing hearts. We found that there were 455 differentially expressed genes between diseased and control LV that were common for both humans and mice (Figure 1A). This list of common differentially expressed genes was then analyzed for similarly dysregulated pathways using gene ontology enrichment analysis. Interestingly, we found multiple occurrences of calcification and fibrosis-related processes among the top 20 enriched pathways. Next, we isolated total RNA from LV of mice with TAC-induced HF that were employed in our previous study (22). We performed miR microarray analysis on these samples, which yielded 158 significantly differentially expressed miRs between HF and control animals. To find miRs that may regulate fibrosis and calcification in the LV, we cross-referenced the fibrosis and calcification-related dysregulated genes found in diseased mouse and human heart RNA-Seq data sets to known targets of the dysregulated miRs in our own microarray analysis data set. This analysis revealed significant downregulation of miR–129-5p and significant upregulation of 4 miR–129-5p targets. Of these miR–129-5p targets, 3 genes are implicated in both fibrosis and calcification processes: *Col1a1*, small leucine–rich proteoglycan *Aspn*, and *Sox9*. Since the role of collagen in cardiac fibrosis has been studied extensively (23) and *Col1a1* was recently shown to be upregulated in context of cardiac OF induced by ischemic injury (11), we focused on ASPN and SOX9.

Of note, another miR, miR–335-3p, was found to be significantly downregulated, and 4 of its fibrosis and calcification-related targets were found to be significantly upregulated in our bioinformatics analysis. However, we were not able to validate downregulated miR–335-3p expression in diseased mouse LV (Supplemental Figure 1A; supplemental material available online with this article; https://doi.org/10.1172/jci.insight.168655DS1).

We next validated downregulation of miR–129-5p and upregulation of its targets Sox9 and Aspn in diseased mouse hearts by quantitative PCR (qPCR). In both the TAC model and chronic Angiotensin II (AngII) infusion model of nonischemic HF, LV expression of miR–129-5p was significantly downregulated in diseased animals compared with their respective controls (Figure 1, B and C). Importantly, we did not find a significant difference in the expression of miR–129-3p between healthy and diseased mouse hearts (Supplemental Figure 1B). While *Aspn* was significantly upregulated both in TAC- and AngII-treated hearts, *Sox9* was significantly upregulated in AngII-treated hearts only (Figure 1, B and C).

Additional analysis of a publicly available single-cell RNA-Seq and FACS database of healthy mouse hearts (24) revealed that fibrosis- and calcification-associated genes *Aspn* and *Sox9* are mainly expressed in CF in mouse hearts (Supplemental Figure 2), supporting the role of CF in both myocardial fibrosis and calcification.

Next, to validate miR–129-5p expression in CF, we employed FISH in healthy mouse hearts and found that miR–129-5p transcripts localize in proximity of CF marker discoidin domain-containing receptor 2 (*Ddr2*) transcripts (Figure 1D). Since FISH probes recognize individually labeled transcripts within a cell, colocalization of 2 transcripts within a cell is demonstrated by the proximity of the 2 transcripts and a nucleus. To ensure miR–129-5p is expressed in CF, we performed microRNA and RNA FACS using fluorescent microRNA and RNA probes (see Methods). This revealed a cell population that was double positive for miR129 and *Ddr2* mRNA (Figure 1E and Supplemental Figure 3), showing that miR–129-5p is indeed expressed in CF, validating our FISH results. Together, these data indicate that miR–129-5p is indeed expressed in CF. However, CF are not the only cell types in the heart expressing miR–129-5p, as miR–129-5p transcripts were also found in close proximity to transcript of the cardiomyocyte marker *Tnnt2* and, to lesser extent, endothelial cell marker *Pecam1* and smooth muscle cell marker *Acta2* (Supplemental Figure 4A).

**Figure 2:**
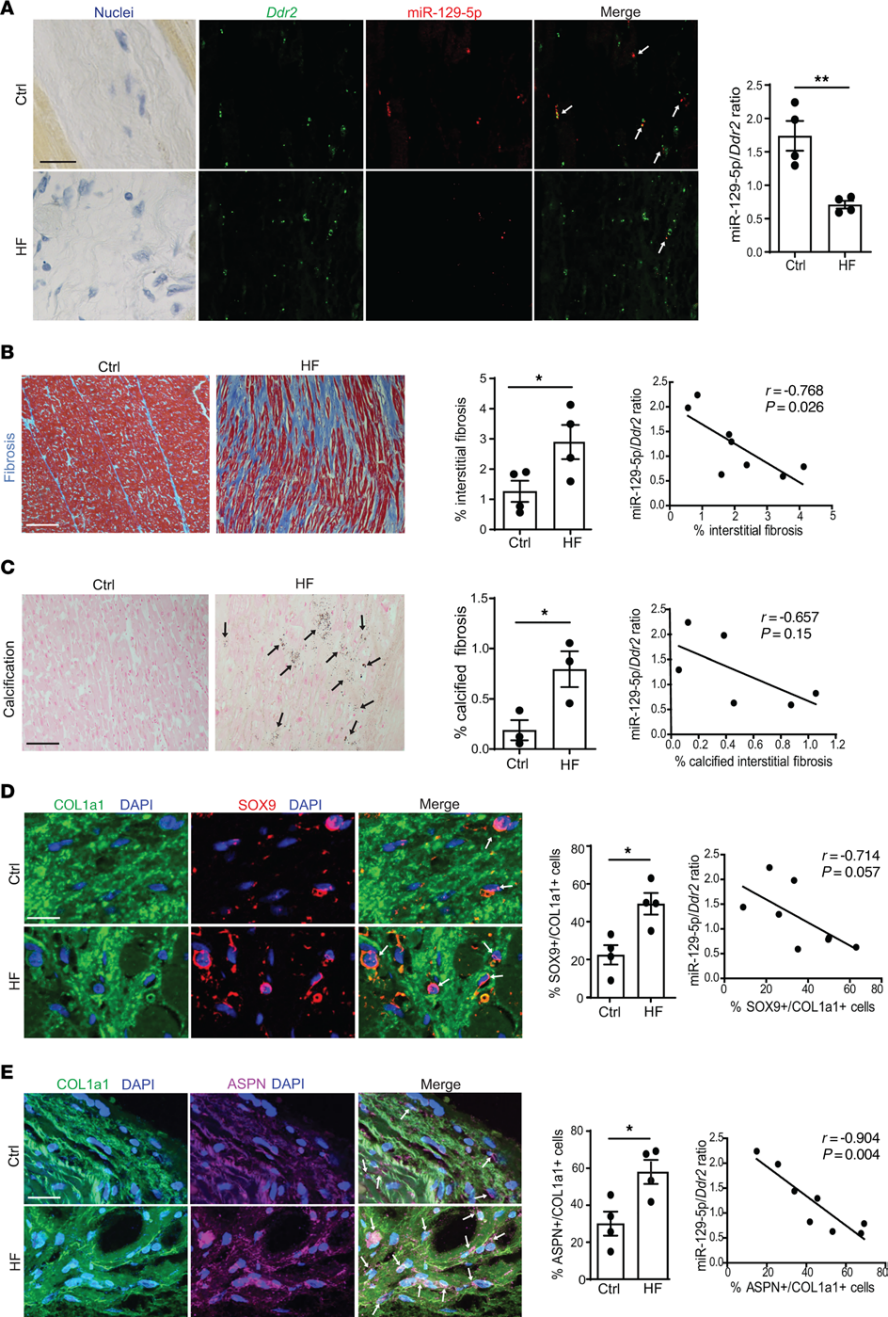
miR–129-5p is downregulated and SOX9 and ASPN are upregulated in CF of patients with HF with LV fibrosis and calcification. (**A**) miR–129-5p (red) expression in CF assessed by FISH with CF marker *Ddr2* (green). Scale bar: 20 μm. Arrows are miR-129-5p+ Ddr2+ cells. (**B**) Interstitial myocardial fibrosis (blue) assessed by Masson’s trichrome staining. Scale bar: 100 μm. (**C**) Calcification (black) in myocardial fibrotic areas assessed by von Kossa staining. Scale bar: 50 μm. Arrows are calcified fibrotic areas. (**D**) Nuclear expression of SOX9 (red) in COL1a1-expressing cells (green) — i.e., CF, assessed by immunofluorescence. Scale bar: 20 μm. Arrows are COL1a1+SOX9+ cells. (**E**) Intra- and extracellular ASPN (pink) expression in COL1a1-expressing cells (green) — i.e., CF, assessed by immunofluorescence. Scale bar: 20 μm. Arrows are COL1a1+ ASPN+ cells. (**B**–**E**) Correlations between the miR–129-5p/*Ddr2* ratio and interstitial fibrosis, calcified interstitial fibrosis, percentage of SOX9-expressing COL1a1^+^ cells, and percentage of ASPN expressing COL1a1^+^ cells in human LV. Data are presented as mean ± SEM; *n* = 3–4 subjects/group. Student’s *t* test. Correlations tested by Pearson’s *r*. *P < 0.05, **P < 0.01. COL1a1: collagen type 1a1, *Ddr2*: discoidin domain-containing receptor 2.

We next validated expression of miR–129-5p and its targets ASPN and SOX9 in LV of patients with chronic HF. Using FISH, we found that miR–129-5p expression in CF is significantly downregulated in LV of patients with HF versus controls, as illustrated by a lower number of miR–129-5p transcripts localizing in proximity of CF marker *Ddr2* transcripts (Figure 2A). Interstitial myocardial fibrosis (Figure 2B) and calcification in fibrotic areas (Figure 2C) were significantly higher in patients with HF versus otherwise-healthy controls. Concomitantly with decreased miR–129-5p expression in CF, we found that the expression of miR–129-5p targets SOX9 (Figure 2D) and ASPN (Figure 2E) in CF was upregulated in LV in patients with HF compared with controls, as assessed by immunofluorescence. Strikingly, in human hearts, the miR–129-5p/*Ddr2* ratio was inversely correlated with LV interstitial fibrosis and expression of ASPN and SOX9 in CF as well as with a trend toward inverse correlation with myocardial calcification in fibrotic areas (Figure 2, B–E).

Together, these data show dysregulated expression of miR–129-5p and its targets ASPN and SOX9 in mouse LV and human CF in the context of nonischemic HF associated with myocardial fibrosis and calcification.

### miR–129-5p regulates CF transition to MF and osteogenic fibroblasts.

**Figure 3:**
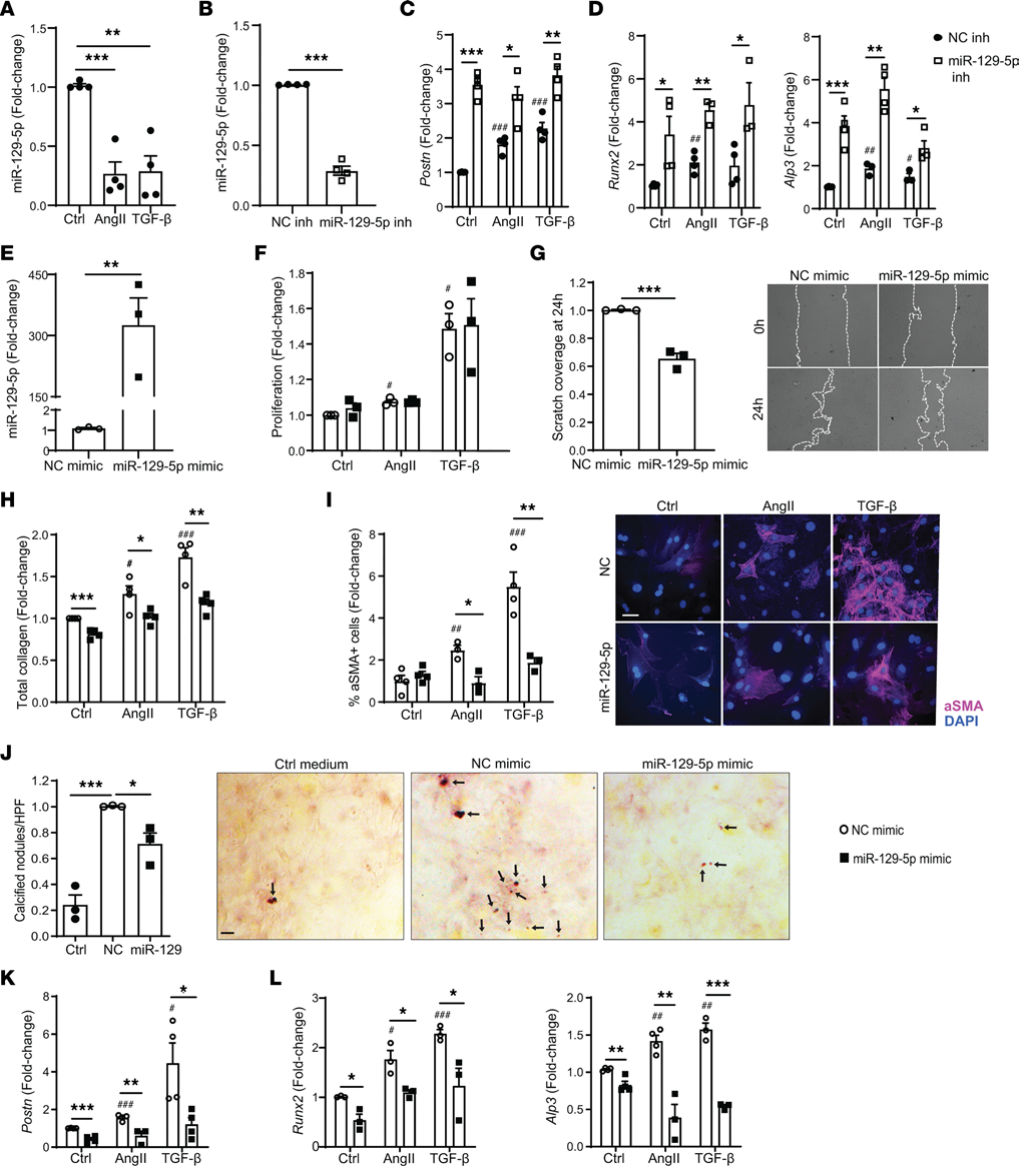
miR–129-5p inhibits both CF-to-MF and CF-to-OF transition. (**A**) miR–129-5p expression in CF upon AngII or TGF-β stimulation as assessed by qPCR. (**B**) miR–129-5p expression in CF upon miR–129-5p inhibition as assessed by qPCR. (**C** and **D**) AngII- and TGF-β–induced expression of *Postn*, *Runx2*, and *Alp3* transcripts in CF upon miR–129-5p inhibition as assessed by qPCR. (**E**) miR–129-5p expression in CF upon miR–129-5p mimic overexpression as assessed by qPCR. (**F**) AngII- and TGF-β–induced CF proliferation upon miR–129-5p mimic overexpression as assessed by CCK8 assay. (**G**) CF migration upon miR–129-5p mimic overexpression as assessed by scratch wound assay. Original magnification ×10. (**H**) AngII- and TGF-β–induced CF collagen production upon miR–129-5p mimic overexpression as assessed by soluble Sirius red assay. (**I**) AngII- and TGF-β–induced αSMA expression in CF upon miR–129-5p mimic overexpression as assessed by immunofluorescence. Scale bar: 10 μm. Original magnification ×40. (**J**) Osteogenic growth medium–induced calcium nodules (dark red, arrows) in CF upon miR–129-5p mimic overexpression as assessed by alizarin red staining. Scale bar: 20 μm. Original magnification ×20. (**K** and **L**) AngII- and TGF-β-induced expression of *Postn*, *Runx2*, and *Alp3* transcripts in CF upon miR–129-5p mimic overexpression as assessed by qPCR. Data are presented as mean ± SEM; *n* = 3–4 independent experiments. Student’s *t* test (**B**, **E**, and **G**) or 1-way ANOVA with Holm-Bonferroni post hoc correction. ^#^*P* < 0.05 versus NC mimic ctrl stimulus, ^##^*P* < 0.01 versus NC mimic ctrl stimulus, ^###^*P* < 0.001 versus NC mimic ctrl; *P < 0.05, **P < 0.01, ***P < 0.001. AngII, Angiotensin II; NC, negative control.

Since miR–129-5p is downregulated in diseased hearts and is associated with myocardial fibrosis and calcification, we next explored the functional role of miR–129-5p in CF-to-MF and CF-to-OF transition. miR–129-5p is known to regulate fibrosis and calcification in other organ systems and cell types (25–28); however, its role in the heart and CF remains unknown. Thus, we assessed miR–129-5p function in primary CF isolated from adult mice. AngII and TGF-β stimulation significantly downregulated miR–129-5p in CF (Figure 3A), similar to our observation in whole LV hearts. Transfection of CF with a miR–129-5p inhibitor resulted in a significant downregulation of miR–129-5p expression (Figure 3B). miR–129-5p inhibition led to enhanced expression of the MF marker *Postn*, expression of the osteogenic transcription factor runt-related transcription factor 2 (*Runx2*), and alkaline phosphatase 3 (*Alp3*) (Figure 3, C and D) at both baseline and upon AngII and TGF-β stimulation. These data suggest that miR–129-5p has a role in maintaining CF quiescence as well as in accentuating MF and OF transition in the presence of injury.

We next assessed the therapeutic potential of miR–129-5p against MF and OF transition. Transfection of CF with miR–129-5p mimic resulted in a significant upregulation of miR–129-5p expression (Figure 3E). While miR–129-5p did not seem to have a significant effect on CF proliferation (Figure 3F), miR–129-5p overexpression did inhibit CF migration (Figure 3G). As expected, AngII and TGF-β stimulation of CF enhanced MF characteristics, including collagen production (Figure 3H), α smooth muscle actin (αSMA) expression (Figure 3I), and *Postn* expression (Figure 3, C and K), all of which were inhibited by miR–129-5p overexpression. Interestingly, AngII and TGF-β stimulation also enhanced osteogenic characteristics in CF, including deposition of calcium nodules (Figure 3J) and expression of the osteogenic transcription factor *Runx2* and *Alp3* (Figure 3, D and L). miR–129-5p overexpression significantly inhibited these OF characteristics. Taken together, these data suggest that miR–129-5p represses both CF-to-MF and CF-to OF transition.

Recently miR–129-5p was shown to inhibit cardiomyocyte hypertrophy (18, 19, 29, 30), and our FISH data show miR–129-5p expression in cardiomyocytes. Cardiomyocytes can affect CF differentiation via paracrine interactions (31); thus, we examined whether miR–129-5p expression in cardiomyocytes may affect CF-to-MF and CF-to OF transition. However, conditioned medium of cardiomyocytes differentially expressing miR–129-5p did not affect expression of MF marker *Postn* or OF marker *Runx2* in CF (Supplemental Figure 4B), indicating that miR–129-5p in cardiomyocytes does not affect CF-to-MF and CF-to-OF transition.

### SOX9 and ASPN are direct targets of miR–129-5p in CF.

**Figure 4:**
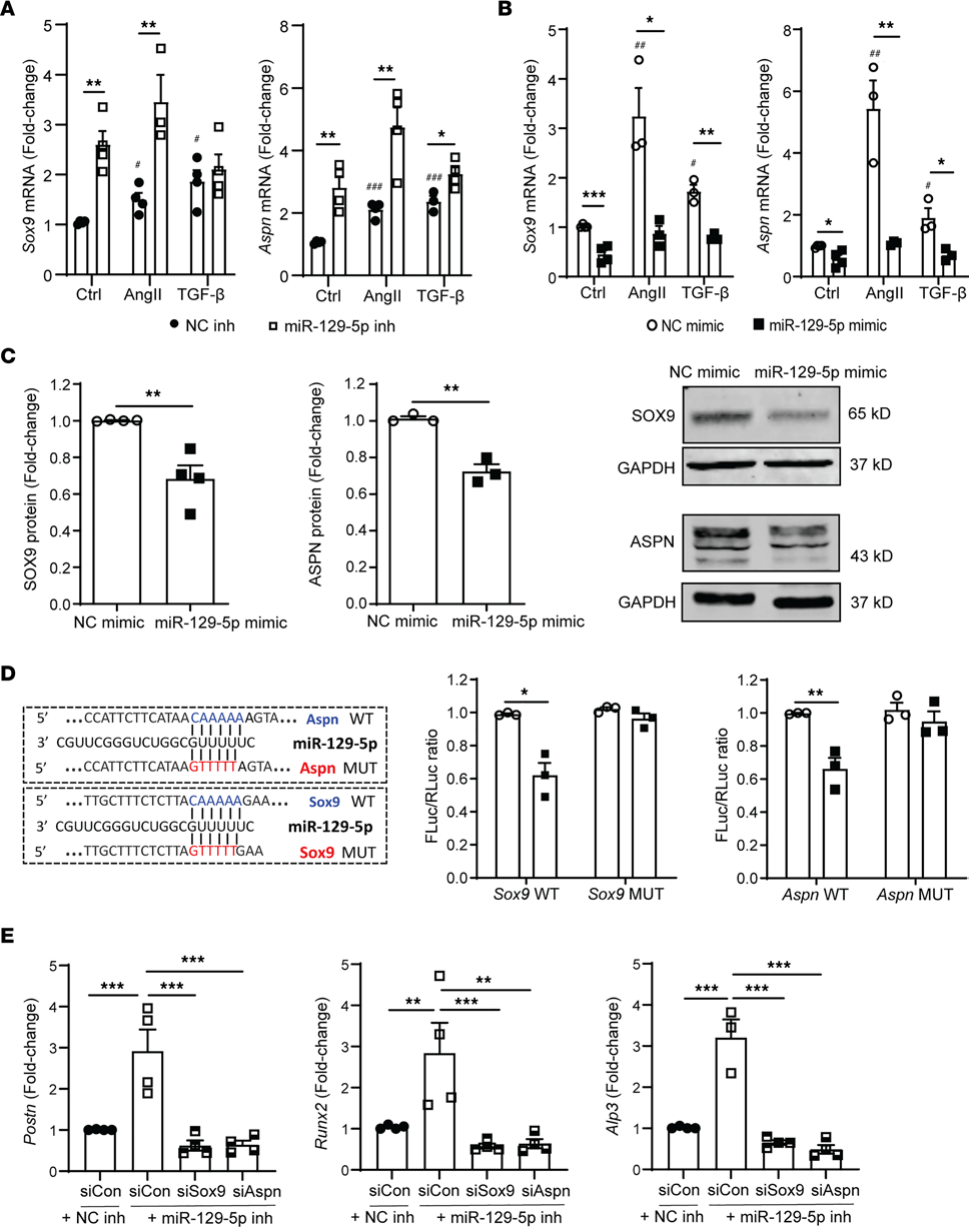
SOX9 and ASPN are direct targets of miR–129-5p. (**A**) AngII- and TGF-β–induced expression of *Sox9* and *Aspn* transcripts in CF upon miR–129-5p inhibition as measured by qPCR. (**B**) AngII- and TGF-β-induced expression of *Sox9* and *Aspn* transcripts in CF upon miR–129-5p mimic overexpression as measured by qPCR. (**C**) SOX9 and ASPN protein expression in CF upon miR–129-5p mimic overexpression with representative Western blots. (**D**) Luciferase activity upon NC or miR–129-5p mimic overexpression in HEK293T cells expressing *Sox9* and *Aspn* constructs containing WT and mutant (MUT) miR–129-5p seed regions. (**E**) Expression of MF marker *Postn* and OF markers *Runx2* and *Alp3* in CF upon miR–129-5p inhibition in presence of control, Sox9, or Aspn siRNA as measured by qPCR. Data are presented as mean ± SEM; *n* = 3–4 independent experiments. Student’s *t* test (**C**) or ANOVA with Holm-Bonferroni post hoc correction.^#^*P* < 0.05 versus NC mimic ctrl stimulus, ^##^*P* < 0.01 versus NC mimic ctrl stimulus,;*P < 0.05, **P < 0.01, ***P < 0.001. AngII, Angiotensin II; NC, negative control.

To further validate our bioinformatics analysis, we next assessed whether SOX9 and ASPN are target genes of miR–129-5p in CF. *Sox9* and *Aspn* gene expression was significantly enhanced in CF transfected with miR–129-5p inhibitor, both at baseline and upon AngII and TGF-β stimulation (Figure 4A). Conversely, *Sox9* and *Aspn* gene and protein expression was significantly downregulated in CF transfected with miR–129-5p inhibitor (Figure 4, B and C). Furthermore, AngII and TGF-β were not able to induce *Sox9* and *Aspn* expression in CF upon miR–129-5p overexpression, while both *Sox9* and *Aspn* were significantly upregulated by AngII and TGF-β in control mimic–treated CF (Figure 4, B and C).

To assess whether *Sox9* and *Aspn* are direct targets of miR–129-5p, we generated luciferase constructs of *Sox9* and *Aspn*, wherein we mutated the miR–129-5p seed region to its complementary sequence (Figure 4D). Luciferase activity in HEK293T cells transfected with WT constructs of both *Sox9* and *Aspn* was inhibited by miR–129-5p mimic overexpression, while there was no inhibitory effect of miR–129-5p on luciferase activity in mutated constructs (Figure 4D).

We next assessed whether SOX9 and ASPN are essential to mediate the effects of miR–129-5p in MF and OF transition. We inhibited miR–129-5p in the presence of SOX9 and ASPN siRNA. Upon SOX9 or ASPN knockdown, miR–129-5p inhibitor was no longer able to promote expression of MF marker *Postn* and OF markers *Runx2* and *Alp3* (Figure 4E).

These data suggest that *Sox9* and *Aspn* are indeed direct targets of miR–129-5p that are necessary for downstream miR–129-5p function in CF-to-MF and CF-to-OF transition.

### SOX9 and ASPN promote CF transition to MF and osteogenic fibroblasts.

**Figure 5:**
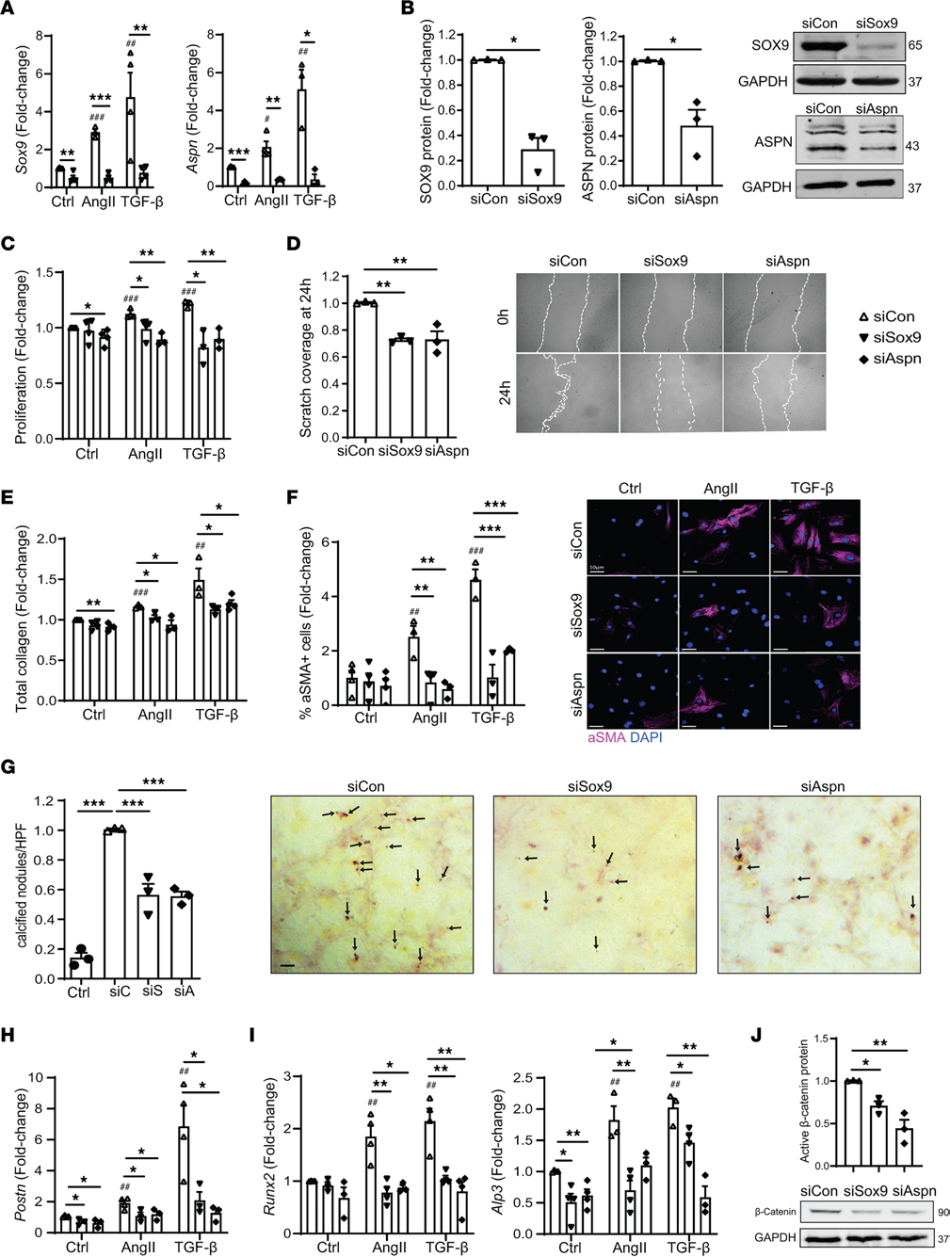
SOX9 and ASPN promote both CF-to-MF and CF-to-OF transition. (**A**) AngII- and TGF-β–induced expression of *Sox9* and *Aspn* transcripts in CF upon Sox9 or Aspn knockdown as assessed by qPCR. (**B**) SOX9 and ASPN protein expression in CF upon Sox9 or Aspn knockdown with representative Western blots. (**C**) AngII- and TGF-β–induced CF proliferation upon Sox9 or Aspn knockdown as assessed by CCK8 assay. (**D**) CF migration upon Sox9 or Aspn knockdown as assessed by scratch wound assay. (**E**) AngII- and TGF-β–induced CF collagen production upon Sox9 or Aspn knockdown as assessed by soluble Sirius red assay. (**F**) AngII- and TGF-β–induced αSMA expression in CF upon Sox9 or Aspn knockdown as assessed by immunofluorescence. Scale bar: 10 μm. (**G**) Osteogenic growth medium–induced calcium nodules (dark red, arrows) in CF upon Sox9 or Aspn knockdown as assessed by alizarin red staining. Scale bar: 20 μm. (**H** and **I**) AngII- and TGF-β–induced expression of *Postn*, *Runx2*, and *Alp3* transcripts in CF upon Sox9 or Aspn knockdown as assessed by qPCR. (**J**) Expression of active β-catenin in CF upon Sox9 or Aspn knockdown as assessed by Western blot. Data presented as mean ± SEM; *n* = 3–4 independent experiments. Student’s *t* test (**B**) or ANOVA with Holm-Bonferroni post hoc correction. ^#^*P* < 0.05 versus NC mimic ctrl stimulus, ^##^*P* < 0.01 versus NC mimic ctrl stimulus, ^###^*P* < 0.001 versus NC mimic ctrl; *P < 0.05, **P < 0.01, ***P < 0.001. AngII, Angiotensin II; NC, negative control.

Since *Sox9* and *Aspn* are direct targets of miR–129-5p, we next assessed the role of these targets in MF and OF transition. While ASPN is known to regulate fibrosis and calcification in different cell types and organs (32–34), and SOX9 is known to regulate MF transition and calcification in different cell types (35–37), thus far, the role of ASPN in CF-to-MF transition and the roles of both SOX9 and ASPN in CF-to-OF transition remain unknown. Knockdown of either SOX9 or ASPN in CF via siRNA resulted in significantly decreased expression on both transcript (Figure 5A) and protein level (Figure 5B). Accordingly, AngII- and TGF-β–induced expression of *Sox9* and *Aspn* in CF was inhibited by their respective siRNAs (Figure 5A). We next assessed the effects of SOX9 and ASPN knockdown on AngII- or TGF-β–induced MF and OF characteristics in CF. We observed significant reductions in proliferation (Figure 5C), migration (Figure 5D), collagen production (Figure 5E), αSMA expression (Figure 5F), calcified nodules (Figure 5G), expression of MF marker *Postn* (Figure 5H), and expression of osteogenic markers *Runx2* and *Alp3* (Figure 5I) in siSox9 and siAspn CF compared with control CF. Taken together, these data suggest that miR–129-5p targets SOX9 and ASPN promote CF-to-MF and CF-to-OF transition.

### SOX9 and ASPN promote β-catenin signaling in CF.

To unravel the mechanism by which SOX9 and ASPN promote CF-to-MF and CF-to-OF transition, we used bioinformatics (STRING protein-to-protein interaction and PANTHER gene set enrichment and gene ontology analyses) to find potential downstream mediators shared between SOX9 and ASPN. We found that both SOX9 and ASPN commonly modulate mediators in the canonical WNT signaling pathway. The primary effector of WNT signaling is β-catenin, and WNT/β-catenin signaling is known to promote MF transition and cardiac fibrosis (38) as well as calcification in vascular smooth muscle cells (39) and osteoblasts (40). Knockdown of either SOX9 and ASPN in CF via siRNA resulted in significantly decreased expression of active β-catenin in these cells (Figure 5J), indicating that Sox9 and Aspn may promote CF-to-MF and CF-to-OF by upregulating β-catenin in CF.

### miR–129-5p overexpression attenuates progression of AngII-induced myocardial fibrosis and calcification and restores cardiac function in mice.

**Figure 6:**
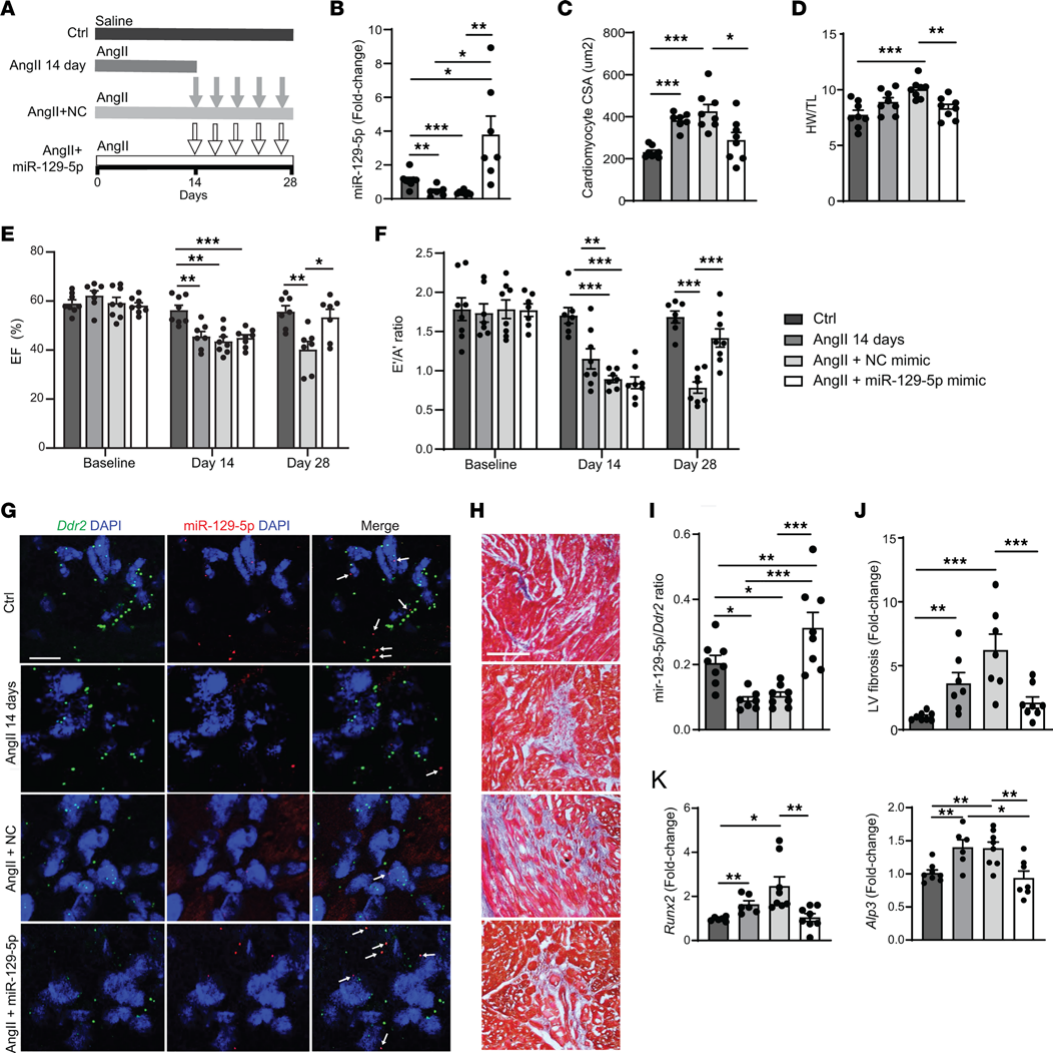
miR–129-5p overexpression attenuates progression of AngII-induced fibrosis and calcification in mice and restores cardiac function. (**A**) Experimental setup for the chronic AngII infusion model. Arrows indicate tail-vein injections of NC mimic or miR–129-5p mimic. (**B**) LV miR–129-5p expression assessed by qPCR. (**C**) Cardiomyocyte hypertrophy assessed by wheat germ agglutinin staining. (**D**) Cardiac hypertrophy assessed by heart weight/tibia length (HW/TL) ratio. (**E**) Systolic function assessed by LV ejection fraction (EF) on serial echocardiography. (**F**) Diastolic function assessed by E’/A’ ratio on serial tissue Doppler imaging. (**G** and **I**) miR–129-5p (red) expression in LV CF assessed by FISH with CF marker ddr2 (green). Scale bar: 10 μm. (**H** and **J**) Interstitial myocardial fibrosis (blue) assessed by Masson’s trichrome staining. (**K**) LV expression of osteogenic markers *Runx2* and *Alp3* transcripts by qPCR. Data are presented as mean ± SEM; *n* = 6–8 mice/group. ANOVA with Holm-Bonferroni post hoc correction; *P < 0.05, **P < 0.01, ***P < 0.001. ddr2, discoidin domain-containing receptor 2; CSA, cross-sectional area; NC, negative control.

Our in vitro data point toward a protective role of miR–129-5p against myocardial fibrosis and calcification. Since myocardial fibrosis and calcification are not always detected early, we aimed to determine the role of miR–129-5p on preexisting myocardial fibrosis and calcification in vivo. Mice were subjected to chronic infusion of AngII (1.5 mg/kg/day) for a total of 28 days. At day 14, mice received either miR–129-5p mimics or negative control mimics i.v. every 3 days for the remainder of the experiment (Figure 6A). A subset of mice was euthanized at day 14 to assess preexisting cardiac pathology prior to starting miR treatment. Expression of miR–129-5p in the whole LV as assessed by qPCR revealed a significant ~3-fold decrease induced by AngII that was restored by miR–129-5p mimic injections (Figure 6B). We observed that miR–129-5p was able to rescue AngII-induced cardiac hypertrophy, as assessed by cardiomyocyte cross-sectional area and heart weight (Figure 6, C and D, and Supplemental Figure 5A). Importantly, before starting miR–129-5p mimic injections, systolic and diastolic function were significantly depressed at 14 days of AngII infusion, and they were both restored by miR–129-5p mimics (Figure 6, E and F, and Supplemental Figure 5, B and C).

We next assessed miR–129-5p expression in CF. We found that AngII significantly decreased miR–129-5p expression in CF ~2-fold already after 14 days, as demonstrated by significantly less miR–129-5p transcripts in proximity of *Ddr2* transcripts, which was restored by miR–129-5p mimic (Figure 6, G and I). Chronic AngII infusion enhanced myocardial fibrosis, which was significantly lower in hearts of miR–129-5p–injected mice (Figure 6, H and J). We furthermore observed that chronic AngII infusion was able to induce expression of osteogenic genes *Runx2* and *Alp3* in the LV, both of which were significantly decreased upon miR–129-5p injections (Figure 6K).

These data show that miR–129-5p mimic treatment in the AngII-induced HF model is not only able to attenuate the progression of preexisting cardiac hypertrophy, myocardial fibrosis, and calcification marker expression but is also able to restore systolic and diastolic dysfunction.

### miR–129-5p attenuates AngII-induced SOX9 and ASPN expression in CF in vivo.

**Figure 7:**
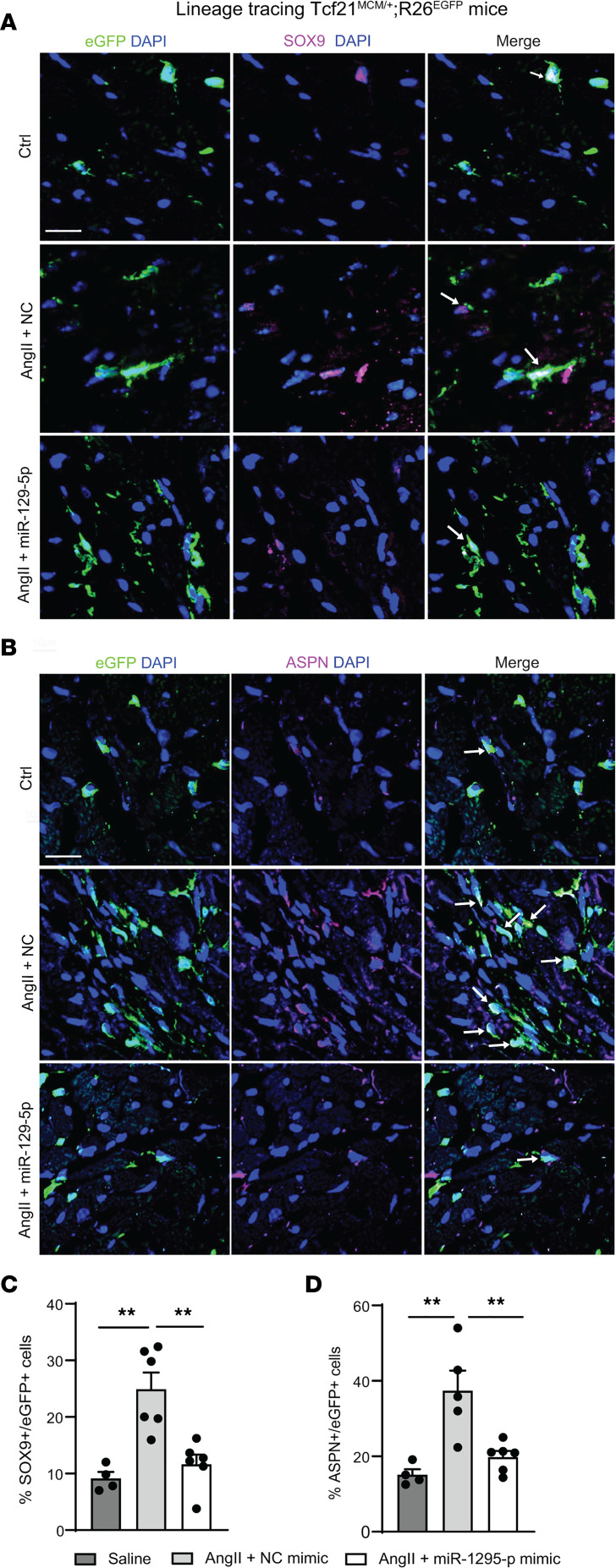
miR–129-5p rescues AngII-induced Sox9 and Aspn expression in CF in vivo. (**A**) Experimental setup for lineage tracing in the chronic AngII infusion model in mice. Arrows indicate tail-vein injections of NC mimic or miR–129-5p mimic. (**B**–**D**) SOX9 and ASPN expression in LV CF of the TCF21 lineage (eGFP^+^) upon chronic AngII infusion and NC or miR–129-5p mimic rescue injections, assessed by immunofluorescence. Scale bar: 10 μm. Data presented as mean ± SEM; *n* = 4–6 mice/group. ANOVA with Holm-Bonferroni post hoc correction. **P < 0.01. eGFP, enhanced green fluorescent protein; NC, negative control; Tcf21, transcription factor 21.

To assess whether the attenuation of myocardial fibrosis and calcification progression by miR–129-5p may be explained by differences in expression of SOX9 and ASPN in CF, we employed lineage tracing of CF of the transcription factor 21 (Tcf21) lineage in mice (Tcf21^MCM/+^;R26^EGFP^ mice). Tcf21^MCM/+^;R26^EGFP^ mice were fed a Tamoxifen diet 4 weeks prior to AngII pump implantation to activate eGFP expression in TCF21-lineage CF. After 14 days of AngII infusion, mice received either miR–129-5p mimics or negative control mimics i.v. every 3 days for the remainder of the experiment (Supplemental Figure 6). We observed that chronic AngII infusion significantly enhanced expression of both SOX9 and ASPN in CF, as indicated by a higher percentage of SOX9^+^eGFP^+^ (Figure 7, A and C) and ASPN^+^eGFP^+^ (Figure 7, B and D) cells in the heart (Figure 7, B and C). Strikingly, miR–129-5p was able to significantly decrease the percentage of both SOX9^+^eGFP^+^ (Figure 7, A and C) and ASPN^+^eGFP^+^ cells (Figure 7, B and D).

Taken together, our data show that, in the context of nonischemic HF, miR–129-5p expression in the LV — and specifically in CF — is downregulated and that miR–129-5p inhibits CF-to-MF and CF-to-OF transition via its targets SOX9 and ASPN and downstream β-catenin signaling. Moreover, overexpression of miR–129-5p via i.v. injection is able to inhibit progression of myocardial fibrosis and calcification and to restore cardiac dysfunction.

## Discussion

Using a bioinformatics and experimental approach in mice and humans, we discovered that miR–129-5p expression in the LV — and specifically in CF — is downregulated in nonischemic HF. We demonstrate that miR–129-5p inhibits CF-to-MF and CF-to-OF transition via its target genes SOX9 and ASPN, which promote β-catenin, thus identifying the common profibrotic and proosteogenic roles of ASPN and SOX9 in CF. Moreover, we demonstrate the therapeutic potential of miR–129-5p against myocardial systolic and diastolic dysfunction, fibrosis, and calcification in vivo (Graphical abstract).

Both myocardial fibrosis and calcification are associated with adverse outcomes in HF (1, 11, 12). Resident CF may transition to both profibrotic MF and procalcifying OF. This CF plasticity is an attractive therapeutic target, as HF, myocardial fibrosis, and myocardial calcification are not often diagnosed early. Since myocardial calcification is often observed in areas of fibrosis, a common upstream mechanism that regulates both CF-to-MF transition and CF-to-OF transition may exist. Here, we show that the common mechanism behind CF-to-MF transition and CF-to-OF transition in nonischemic HF comprises the miR–129-5p/SOX9 and miR–129-5p/ASPN axes. Recently miR–129-5p was shown to inhibit cardiomyocyte hypertrophy (18, 19, 29, 30). Although our study confirms the role of miR-129 in regulating cardiac hypertrophy as well, ours is the first study to our knowledge to investigate the role of miR–129-5p in regulating fibrosis and calcification in the heart by targeting CF and not cardiomyocytes. We additionally show that miR–129-5p expression in cardiomyocytes does not affect CF differentiation and that analysis of online-available single-cell RNA-Seq and FACS data shows that miR–129-5p targets Sox9 and Aspn are mainly expressed in CF in the heart. These data strongly support the importance of the role of miR–129-5p in CF in myocardial fibrosis and calcification. Furthermore, regardless of the role of miR–129-5p, we demonstrate that ASPN promotes CF differentiation, that SOX9 promotes CF-to-OF differentiation, and that AngII and TGF-β induce an osteogenic phenotype in CF.

miR–129-5p has previously been shown to inhibit fibrosis in other organs, including liver (26, 41, 42), kidney (27), and skin (43). Additionally, miR–129-5p was reported to inhibit osteogenic differentiation and calcification in MC3T3 osteoblastic cells (25), mesenchymal stem cells (44), adipose-derived stem cells (28), and renal fibroblasts (45). Interestingly, however, miR–129-5p was also shown to promote osteogenic differentiation in bone marrow–derived stem cells (46). Together, these reports of the antifibrotic and antiosteogenic roles of miR–129-5p in other cell types support our exploratory bioinformatics approach on discovering miR–129-5p as a differentially expressed miR in myocardial fibrosis and calcification in the diseased LV.

Our bioinformatics analysis revealed that expression of both miR–129-5p targets ASPN and SOX9 was enhanced in mouse and human LV in nonischemic HF. We furthermore show the functional role of Aspn in promoting CF-to-MF transition. We show, for the first time to our knowledge, that ASPN and SOX9 both promote CF-to-OF transition. Supporting our data, upregulation of both ASPN and SOX9 has previously been reported upon ischemic injury in infarcted pig and mouse hearts, respectively (35, 47). ASPN was shown to enhance expression of fibrosis-related genes in H9C2 myoblasts (34) but also to repress MF characteristics in keloid scar dermal fibroblasts (48). SOX9 has already been reported to promote CF proliferation and migration in vitro (36), and accordingly, both full body SOX9-deficient and CF-specific SOX9-deficient mice had attenuated cardiac dysfunction and fibrosis after myocardial infarction (35, 36). Supporting our findings on CF-to-OF transition and calcification, ASPN has been shown to enhance calcification in osteoblasts, dental pulp stem cells, and dermal fibroblasts (32, 33, 48), while SOX9 was reported to enhance calcification in vascular smooth muscle cells (37, 49) and human aortic valve interstitial cells (50). On the other hand, both ASPN and SOX9 were also reported to inhibit calcification in aortic valve interstitial cells and heart valves (51, 52). ASPN is a small leucine–rich proteoglycan, a class of proteins able to interact with different components of the ECM, cell surface receptors, and growth factors (53). As a transcription factor, SOX9 may bind clusters of different enhancers to regulate specific gene expression (54). Therefore, the differential effects of ASPN and SOX9 on calcification may be cell type and context dependent. In vitro, we show that, following Sox9 or Aspn knockdown, miR–129-5p inhibitor is no longer able to significantly promote expression of MF and OF markers in CF, suggesting that SOX9 and ASPN are indeed essential for miR–129-5p–dependent CF activity. Due to technical limitations in these experiments, we transfected all siRNAs and miR inhibitors together, rather than inhibiting miR–129-5p in cells deficient for SOX9 and ASPN. As such, we cannot assess the exact proportion of the CF miR–129-5p effects that SOX9 and ASPN contribute to. Also, to which extent ASPN and SOX9 account for miR–129-5p effects in CF differentiation in vivo remains to be elucidated. *Sox9* and *Aspn* are not the exclusive targets of miR–129-5p. As a miR, miR–129-5p has numerous target genes with roles in pathways controlling cardiac remodeling and HF. As we mainly focus on unraveling common mechanisms in myocardial fibrosis and calcification, we performed our bioinformatic analysis to discover miRs and target genes that regulate both fibrosis and calcification together. However, other miR–129-5p target genes that do not regulate both fibrosis and calcification together, but regulate either fibrosis or either calcification separately, may still exert their effects and account for part of the protective miR–129-5p effect in the heart.

The central role of MF in cardiac fibrosis has been evident. However, the role of OF in myocardial calcification is emerging. We show for the first time to our knowledge that both AngII and TGF-β can induce CF-to-OF transition in vitro and that chronic AngII infusion in C57BL/6J mice induces expression of calcification markers in the LV and enhances calcium deposits in myocardial fibrotic areas. Recently, it was shown that CF can adopt osteogenic cell fates and promote dystrophic myocardial calcification after ischemic injury by left anterior descending coronary artery ligation and necrotic injury after cryoinjury (11). Mechanistically, it was shown that CF express the pyrophosphate-generating enzyme Ectonucleotide pyrophosphatase/phosphodiesterase-1 (ENPP1) upon cardiac cryoinjury to promote calcification (11). However, upstream mechanisms regulating OF phenotypes in nonischemic HF-associated myocardial calcification remain unknown. Our data are in line with reports of TGF-β promoting calcification in dermal fibroblasts and aortic valve interstitial cells (55, 56) and with reports of AngII enhancing calcification in aortic valve MF and vascular smooth muscle cells (57, 58). While our bioinformatics approach is based on RNA-Seq and miR microarray data sets of the nonischemic model of TAC-induced, cardiac pressure overload–induced HF, we were not able to validate SOX9 as a dysregulated miR–129-5p target gene in TAC LV by qPCR. Since both SOX9 and ASPN were validated to be upregulated in LV upon chronic AngII infusion, we pursued this in vivo model to further investigate the role of miR–129-5p in myocardial fibrosis and calcification in mice. We believe that sustained AngII infusion with more advanced myocardial fibrosis and HF would further progress myocardial calcification to more severe forms. Our findings are furthermore supported by reports of the ability of AngII receptor blockers to attenuate vascular calcification in rodents (59, 60) in rodents. Moreover, emerging reports show that, in patients with aortic stenosis, the use of AngII receptor blockers results in slower progression of aortic valve calcification (61).

We report that CF-specific miR–129-5p expression is significantly downregulated in patients suffering from chronic HF with LV myocardial fibrosis and calcification. However, with a limited number of patients with HF and controls, we furthermore show a negative correlation between CF miR–129-5p expression and myocardial fibrosis, with a trending negative correlation between CF miR–129-5p expression and myocardial calcification. In line with our data, downregulated miR–129-5p expression has been reported in serum from patients with chronic HF (62, 63). miR–129-5p expression was shown to correlate with improved ejection fraction, and lower miR–129-5p levels were predictive of poor prognosis (63). Whether serum miR–129-5p levels could also be predictive for the extent of myocardial fibrosis and calcification remains to be elucidated.

Lastly, we demonstrate the therapeutic potential of miR–129-5p since systemic delivery of miR–129-5p mimic in an AngII HF model was able to attenuate the further progression of preexisting cardiac hypertrophy, myocardial fibrosis, and calcification marker expression; it was also able to restore systolic and diastolic function that was dysregulated by AngII. We furthermore show by lineage tracing that miR–129-5p mimics are able to attenuate AngII-induced expression of pro-MF and pro-OF mediators ASPN and SOX9 in CF. We cannot exclude that miR–129-5p in other cardiac types may partially contribute to the observed restored cardiac function. We recognize the interplay between the inflammatory and fibrotic response in HF as well as the role of cardiomyocytes in improved systolic and diastolic function. Indeed, our FISH data show miR–129-5p expression in cardiomyocytes in mouse hearts as well, and we observed attenuated cardiac hypertrophy after miR–129-5p mimic injections in mice. This is in line with recent reports of miR–129-5p repressing hypertrophy, oxidative stress, autophagy, and myofibrilogenesis in cardiomyocytes (18, 19, 62, 64, 65). However, we also show that miR–129-5p expression in cardiomyocytes does not seem to affect MF and OF transition, as conditioned medium from miR–129-5p overexpressing cardiomyocytes does not affect *Postn* and *Runx2* expression in CF. Additionally, ViewRNA FISH showed minimal expression of miR–129-5p in smooth muscle cells and endothelial cells in the LV and single-cell RNA-Seq and FACS bioinformatics revealed that miR–129-5p targets SOX9 and ASPN are mainly expressed in CF. Therefore, we believe that the antifibrotic and anticalcification effects we observed in vivo were mainly mediated by miR–129-5p/SOX9 and miR–129-5p/ASPN signaling in CF. Future studies will assess the specificity of miR–129-5p in CF, as a limitation of our study is that we did not use CF-specific miR–129-5p–KO or –overexpression mouse models or CF-specific delivery of miR–129-5p mimics. However, any non-CF off-target effect that systemic miR–129-5p delivery may have still produces beneficial outcomes regarding myocardial fibrosis, calcification, systolic, and diastolic function, highlighting the therapeutic potential of miR–129-5p.

Taken together, our work provides insights into the pathogenic mechanisms and possible therapeutic avenues in myocardial fibrosis and calcification in the context of nonischemic HF by targeting CF functional plasticity.

## Methods

Supplemental Methods are available online with this article.

### Bioinformatics.

Online available RNA-Seq data sets of human (20) (GSE135055; 21 patients with HF, 9 healthy donors) and mouse (21) HF (GSE112055; 5 sham, 5 TAC) from the NCBI GEO database were used. These data sets were reanalyzed from the expression matrices supplied in the respective supplementary files using the DESeq2 R package (66). Human and mouse statistical results were then cross-referenced to obtain a list of mouse genes differentially expressed relevant to the human disease. This list was defined by an absolute fold change > 1.4 and *P* < 0.05 in mice and an absolute fold change > 1.4 in humans. The list of differentially expressed genes was next used to investigate pathways similarly dysregulated in humans and mice using the web-based gene ontology enrichment tool PANTHER (67). This analysis revealed multiple fibrosis and calcification-related processes among the top 20 enriched pathways. LV tissue of sham and TAC mice (4 sham, 5 TAC) was sent to Ocean Ridge Biosciences for miR microarray screening using proprietary single channel arrays (MirBASE 16.0 MiR Array, Ocean Ridge Biosciences). The hearts of these mice were employed in our previous study (22). Data can be accessed in GSE225067. Differentially expressed miRs between sham and TAC were defined by a log fold change (logFC) >0.5 and <0.5 and FDR <0.05. Next, targets of the differentially expressed miRs were identified using GSEA miRNA gene sets (c3.mir.v7.2.symbols) (68, 69). Finally, targets of the differentially expressed miRs were cross-referenced with the differentially expressed genes that are part of fibrosis and calcification processes, as found in the human and mouse RNA-Seq databases.

An online-available web server (https://tabula-muris.ds.czbiohub.org/) was employed to analyze gene and protein expression in a publicly available single-cell RNA-Seq and FACS data set of healthy mouse hearts (7 mice) (24).

### Human cardiac tissue.

Formalin-fixed paraffin-embedded, human cardiac tissue sections were obtained from the Department of Pathology at UCLA. Patients with chronic HF with LV fibrosis, calcification and expression of miR–129-5p, SOX9, and ASPN were compared with control subjects not suffering from HF. Patient characteristics are displayed in Supplemental Table 2.

### Animals.

Male 8- to 10-week-old C57BL/6J mice were purchased from the Jackson Laboratory. For lineage tracing of CF, we employed mice that express the tamoxifen-regulated MerCreMer under control of the TCF21 gene locus, along with a loxP site–dependent EGFP reporter in the ubiquitous Rosa26 gene locus (TCF21^MCM/+^;R26^EGFP^) (13). We received a breeding pair from Yibin Wang (Department of Anesthesiology & Perioperative Medicine, David Geffen School of Medicine, UCLA), after which we established our own colony. Male 4- to 6-week-old TCF21^MCM/+^;R26^EGFP^ mice were used for experiments.

### CF isolation.

Protocol adapted from Melzer et al. (70). Male, 8- to 10-week-old C57BL/6J mice were euthanized under deep isoflurane anesthesia (4%), followed by cervical dislocation. Hearts were quickly excised, and atria were trimmed off. Ventricles were next minced into ~1 mm^2^ pieces and incubated in 100 units/mL Collagenase Type II (Worthington) in PBS at 37°C under constant agitation until all tissue was digested or for a maximum of 40 minutes. Next, digested tissue was passed through a 100 μm cell strainer (Thermo Fisher Scientific) and cells were centrifuged (room temperature, 500 g for 10 minutes). To select for CF, cells were suspended in DMEM-F12 + 10% FCS + 1% antibiotic/antimycotic + 10 ng/mL b-FGF (all from Thermo Fisher Scientific) and preplated on 0.1% gelatin-coated culture dishes for 3 hours at 37°C and 5% CO_2_. Next, nonadherent cells were removed and CF were cultured until sufficient confluence was reached, while medium was refreshed daily. Experiments were performed with passage 2 and 3 CF.

### In vivo AngII model.

Osmotic pumps (ALZet, model 1004) containing 1.5 mg/kg/day AngII or saline were implanted in male 8- to 10-week-old C57BL/6J mice under isoflurane anesthesia (4% induction, 1.5%–2% maintenance). Buprenorphine (0.1 mg/kg s.c., every 12 hours for 24 hours) was used as analgesic. For lineage-tracing experiments, 4- to 6-week-old male Tcf21^MCM/+^;R26^EGFP^ mice were fed a tamoxifen diet (Envigo, TD.130860) for 4 weeks, followed by a week of acclimatization prior to pump implantation. After 14 days of AngII infusion, a subset of mice was sacrificed, and the remaining mice were subjected to tail-vein injections containing 10 nmol miR–129-5p mimic or negative control mimic (miRVana mimic In Vivo Ready, Thermo Fisher Scientific, catalogs 4464061 and 4464066) approximately every 3 days, for a total of 5 injections. At day 28 of AngII infusion, mice were sacrificed under deep isoflurane anesthesia by cardiac blood draw, followed by removal of the heart. Tissues were snap-frozen in liquid nitrogen or fixed in 4% paraformaldehyde (Thermo Fisher Scientific).

### Echocardiography.

During the experiment, transthoracic echocardiography was performed using the Vevo 2100 Imaging System (Visual Sonics) under isoflurane anesthesia (4% induction, 1.5%–2% maintenance) at baseline, 14 days, and 28 days of AngII infusion. LV systolic and diastolic function were assessed via B-mode, M-mode, and tissue Doppler imaging.

### Tissue preparation for histology.

Mouse hearts were fixed in 4% paraformaldehyde (Thermo Fisher Scientific), immersed in 20% sucrose (MilliporeSigma), and embedded in OCT compound (Sakura Tissue-Tek). Mouse and human cardiac tissue was cut to 10 μm sections for in situ hybridization and 7 μm sections for histological and immunofluorescence staining. Images were acquired using a Nikon A1 confocal microscope.

### Masson trichrome staining.

The UCLA Pathology Core performed Masson Trichrome staining on human cardiac tissue. Mouse tissue was stained using the Masson Trichrome Kit (MilliporeSigma, HT15) according to manufacturer instructions. Images were made using a Nikon A1 confocal microscope. For human cardiac tissue, interstitial fibrosis as a proportion of the entire tissue section and, for mice, interstitial fibrosis as a proportion of the entire LV was measured using ImageJ software (NIH).

### von Kossa staining.

Myocardial calcification in human cardiac tissue sections was assessed using the von Kossa staining kit (Abcam, ab150687) according manufacturer instructions using a UV for 2 hours. Calcified fibrotic area was measured using ImageJ and was calculated as a proportion of the total fibrotic area.

### Wheat germ agglutinin staining.

Heart sections were stained with Alexa Fluor 594–conjugated wheat germ agglutinin (Molecular Probes) and mounted in Fluoromount G with DAPI (Thermo Fisher Scientific, 00-4959-52). Transversely cross-sectioned cardiomyocytes with thin cell borders and nuclei close to the cell center were used for assessment of cross-sectional area (Image J).

### FISH.

The ViewRNA Tissue Kit (Thermo Fisher Scientific, 19931 and 19932) was employed according to manufacturer’s instructions. The following ViewRNA probes were used for detection: miR–129-5p (VM1-10479-VCP), human *Ddr2* (VA6-3168788-VC), and mouse *Ddr2* (VB6-12897-VC). Tissue slides were mounted with Advantage Mounting Medium (Innovex, NB300) and imaged on a Nikon confocal microscope. Single transcripts of miR–129-5p and Ddr2 were counted and calculated as a miR–129-5p/*Ddr2* ratio in 5 fields per tissue section

### Flow cytometry in situ hybridization.

A cardiac cell suspension was prepared using a protocol adapted from Forte et al. (71) Briefly, male 10- to 12-week-old C57BL/6J mice were euthanized under deep isoflurane anesthesia (4%), followed by cervical dislocation. Ventricles were excised and minced into ~1 mm^2^ pieces and incubated in 100 units/mL Collagenase Type II (Worthington) in PBS at 37°C in a water bath under constant agitation 2 times for 15 minutes. Next, digested tissue was collected in 2%FBS in PBS and passed through a 100 μm cell strainer (Thermo Fisher Scientific), and cells were centrifuged (room temperature, 500 g for 10 minutes). RBCs were lysed with ACK Lysing Buffer (Thermo Fisher Scientific). The PrimeFlow RNA Assay (Thermo Fisher Scientific, 88-18005) was employed according to manufacturer instructions. A fixable viability dye (L34963) and probes for *Rpl13a* (VB6-15315), *Ddr2* (VB10-3282095), and miR–129-5p (VM1-10479) were used. Samples were next analyzed on an Attune NXT Flow Cytometer (Thermo Fisher Scientific) and FlowJo Software.

### Immunofluorescence.

After removing paraffin or OCT compound, cardiac tissue sections were boiled in citrate buffer for 30 minutes as antigen retrieval and blocked with either 5% normal donkey or 5% normal goat serum (Jackson ImmunoResearch, 017-000-121 and 005-000-121). Antibodies against SOX9 (1:200, MilliporeSigma, AB5535), ASPN (1:100, Novus Biologicals, NB100-1514), COL1a1 (1:200, Santa Cruz Biotechnology Inc., sc-293182), or GFP (1:200, Abcam, ab13970) were incubated overnight at 4°C. Secondary Alexa Fluor antibodies (1:1,000, Invitrogen, catalogs A21245, A11001, A102524, A11055; Thermo Fisher Scientific, A-11039) were incubated for 1h at room temperature. Tissue slides were mounted in Fluoromount G with DAPI (Thermo Fisher Scientific, 00-4959-52) and imaged using a Nikon A1 confocal microscope.

For human cardiac LV sections, SOX9^+^COL1a1^+^ and ASPN^+^COL1a1^+^ cells were counted and calculated as the proportion of total COL1a1^+^ cells in 7–8 random fields per tissue section. For mouse lineage tracing experiments, cells double positive for SOX9^+^eGFP^+^ and ASPN^+^eGFP^+^ cells were counted and calculated as the proportion of total eGFP^+^ cells in 5 random fields per cardiac LV tissue section.

For cell experiments, CF were grown on coverslips and, after experiments, were fixed in 4% paraformaldehyde (Thermo Fisher Scientific), permeabilized with 10% Triton and blocked with 5% normal goat serum. Alexa Fluor 647–conjugated αSMA antibody (1:500, Novus Biologicals, NBP2-34522AF647) was incubated overnight at 4°C, and sections were mounted with Fluoromount G with DAPI. Cells positive for αSMA as a proportion of total cell number was quantified in 5 random fields per coverslip per experimental replicate.

### miRNA and siRNA transfections.

Negative controls, miR–129-5p mimic, or miR–129-5p inhibitor (miRVana, ThermoFisher Scientific, catalogs 4464058, 4464066, 4464076, 4464084; 40 nM) or siRNA targeting mouse Sox9 and Aspn or scrambled control (SMARTPOOL, Dharmacon, catalogs L-055742-01-0005, L-059108-01-0005, and D-001810-10-05; 40 nM) were transfected into CF using Lipofectamine RNAiMAX (Thermo Fisher Scientific) in Optimem medium (Thermo Fisher Scientific) for 6 hours, after which medium was replaced with DMEM-F12 + 0.5% FCS + 1% antibiotic/antimycotic. Experiments were performed 24 hours after transfection. CF were stimulated with AngII (1 μM; Thermo Fisher Scientific, A9525) or TGF-β (10 ng/mL; PeproTech, 100-21) for indicated time points.

### RNA isolation and qPCR.

Total RNA was isolated from CF or cardiac tissue powder using Trizol (Thermo Fisher Scientific) according to manufacturer instructions. For mRNA qPCR, reverse transcription was performed using the High Capacity cDNA Reverse Transcription kit (Thermo Fisher Scientific, 4368814) and qPCR was performed using Power Up SYBR Green Master Mix (Thermo Fisher Scientific, A25779) on a Bio-Rad CFX Connect PCR detection system. Primers are listed in Supplemental Table 1. For miRNA qPCR, Taqman RT kit (Thermo Fisher Scientific, 4366596), Taqman assays (Thermo Fisher Scientific, 4427975; Assay IDs 001973, 000590, 002185), and Taqman Universal Master Mix (Thermo Fisher Scientific, 4440040) were employed.

### Protein extraction, SDS-PAGE, and Western blotting.

Whole-cell protein lysates were prepared using RIPA lysis buffer (50 mM NaCl, 50 mM Tris [pH 8], 1% NP-40, 0.5% sodium deoxycholate, and 0.1% SDS; MilliporeSigma) containing protease and phosphatase inhibitors (Roche, 04-906-845-001 and 118-3615-3001). Proteins were diluted in 4***×*** Laemmli sample buffer (Bio-Rad, 161-0747), boiled, and separated on 10% gels by SDS-PAGE. Next, proteins were transferred onto nitrocellulose membranes (Bio-Rad, 170-4270) using semidry blotting (TransBlot Turbo System, Bio-Rad). After transfer, membranes were blocked with 5% BSA (MilliporeSigma, A9647) and incubated with antibodies directed against Sox9 (1:500, MilliporeSigma, AB5535), Aspn (1:500, Novus Biologicals, NB100-1514), active β-catenin (1:500, Cell Signaling Technology, 19807), and GAPDH (1:10.000, Cell Signaling Technology, 2118). IRDye-conjugated secondary antibodies (1:10,000, LI-COR, catalogs 32210, 68070, and 32214) were used for detection, and blots were scanned using the LI-COR Odyssey Infrared Imaging System. Band intensity was quantified using Image Studio Lite Version 5.2.

### Cell proliferation assay.

CF were seeded in a 96-well plate (5,000 cells/well), transfected accordingly, and treated with 1 μM AngII, 10 ng/mL TGF-β, or vehicle. CCK8 assay (Dojindo Laboratories, CK04) was performed 24 hours after stimulation, according to the manufacturer’s protocol.

### Scratch wound assay.

After transfection, CF were grown to ~90% confluence, and a scratch was made using a p1000 pipette tip after which the culture medium was refreshed. Pictures of the entire scratch were made using a Zeiss Axiovert microscope at t = 0 hours and t = 24 hours after the scratch was made. Using ImageJ (NIH), the surface area of the entire scratch wound at t = 0 hours and t = 24 hours was measured, and scratch wound coverage at 24 hours was calculated.

### Soluble Sirius Red assay.

Collagen content in CF was measured as described previously (72). Briefly, after transfection, CF were stimulated with AngII (1 μM) or TGF-β (10 ng/mL) for 72 hours. Afterward, the culture medium was discarded and the cells were fixed with 4% paraformaldehyde (Thermo Fisher Scientific). To stain the collagen, cells were incubated with 0.1% Sirius Red dye (MilliporeSigma, 365548) in 0.01M HCl for 1 hour at room temperature. After extensive washing with 0.01M HCl, the dye was dissolved in 0.01M NaOH and absorbance was measured at OD 550 in a microplate reader (Synergy H1, BioTek). OD values were compared with a gelatin (MilliporeSigma) standard curve

### Alizarin red assay.

After transfections, CF were grown in osteogenic differentiation medium (11) (α-MEM [Thermo Fisher Scientific] + 10% FBS, 10 nM dexamethasone [MilliporeSigma, D4902], 20 mM β-glycerol phosphate [MilliporeSigma, G9422], and 50 μM L-ascorbic acid [MilliporeSigma, A4403]) for 7 days. Next, CF were fixed with 4% paraformaldehyde (Thermo Fisher Scientific) and were stained with alizarin red (MilliporeSigma, TMS-008-C) for 1 hour at room temperature. The dye was carefully rinsed off, and images were made using a Zeiss Axiovert microscope. Calcium nodules were counted in 5–10 fields per well per experimental replicate.

### Luciferase assays.

Luciferase constructs of human Sox9 and Aspn 3′UTR were purchased from Geneocopeia (HmiT127361-MT06, HmiT127360-MT06). Using site-directed mutagenesis, the seed region of miR–129-5p (CAAAAA) in these constructs was mutated into the complementary sequence (GTTTTT). Mutations were generated using the Q5 Site-Directed Mutagenesis Kit (New England Biolabs, E0554) and confirmed by sequencing (Laragen). The following primers were used: *Aspn* forward primer: 5′-TTTGTAAGATATTCGGTATTTAACAC-3′; *Aspn* reverse primer: 5′-AAACTTATGAAGAAAGGAATGGTC-3′; *Sox9* forward primer: 5′-TTTAAATTCTTTTTTTTTTTTTTTTCCAATTTTAC-3′; *Sox9* reverse primer: 5′-AAACTTTTAATTAAAACCTCTCCTTAC-3′.

HEK293T cells were seeded onto 0.1% gelatin in a 24 wells plate in DMEM + 10%FCS (Thermo Fisher Scientific). Using Lipofecatimne 2000 (Thermo Fisher Scientific) and Optimem medium, 100 ng/well of WT or MUT *Aspn* or *Sox9* constructs, together with 40 nM miR–129-5p mimic or negative control mimic, was transfected for 24 hours. Luciferase assay was performed using the Luc-Pair Duo-Luciferase Assay Kit 2.0 (Geneocopeia, LF001) according to manufacturer’s protocol on a Synergy H1 microplate reader.

### Statistics.

Data distribution was tested with the Kolmogorov–Smirnov normality test. Significant outliers were tested with Grubb’s test. Data are presented as mean ± SEM and tested with 2-tailed Student’s *t* test (2 groups) or 1-way ANOVA with Holm-Bonferroni post hoc correction (>2 groups). Correlation was tested using Pearson’s *r*. A *P* value less than 0.05 was considered significant. All analyses were performed using GraphPad Prism 9 software.

### Study approval.

IRB-exempt, deidentified, formalin-fixed paraffin-embedded, human cardiac tissue sections were obtained from the Department of Pathology at UCLA. Tissues were collected in accordance with the Declaration of Helsinki. The Institutional Animal Research Committee approved all animal procedures (ARC-2010-045) according to current NIH guidelines.

## Author contributions

LM and ME contributed conceptualization; LM contributed formal analysis; LM contributed validation; LM, LA, GR, ML, NS, WS, S Sarji, JH, S Sharma, and GF contributed investigation; LM and GR contributed visualization; LM, LA, ME, GR, ML, NS, WS, S Sarji, JH, S Sharma, and GF contributed methodology; LM and ME contributed writing of the original draft; LM, LA, GR, ML, NS, WS, S Sarji, JH, S Sharma, RO, GF, and ME contributed review and editing of the manuscript; RO and ME contributed resources; and ME contributed supervision.

## Supplementary Material

Supplemental data

## Version 1 05/08/2023

Electronic publication
