# Supplementary material for: Myocardial fibrosis and calcification are attenuated by microRNA–129-5p targeting Asporin and Sox9 in cardiac fibroblasts
Source: JCI Insight. 2023 May 8;8(9):e168655. doi: 10.1172/jci.insight.168655 (PMC10243800; doi:10.1172/jci.insight.168655)
Supplement: Supplemental data [file jciinsight-8-168655-s123.pdf]

SUPPLEMENTAL MATERIAL

SUPPLEMENTAL METHODS

Fluorescence in situ hybridization (FISH)

The ViewRNA Tissue Kit (Thermo Fisher #19931 & #19932) was employed according to manufacturer's instructions. The following ViewRNA probes were used for detection: miR-129-5p (VM1-10479-VCP), mouse Tnnt2 (VB6-3202146), mouse Pecam1 (VB6-12921), mouse Acta2 (VB6-12923). Tissue slides were mounted with Advantage Mounting Medium (Innovex, #NB300) and imaged on a Nikon confocal microscope.

Conditioned medium experiments

H9C2 cardiac myoblasts were transfected with negative control or miR-129-5p mimics (miRVana, ThermoFisher #4464058, #4464066, 40nM) using Lipofectamine RNAiMAX (ThermoFisher) in Optimem medium (ThermoFisher) for 6h, after which medium was replaced with DMEM+1%FCS+ 1%antibiotic/antimycotic. After 24h, conditioned medium from H9C2 cells was transferred to mouse primary cardiac fibroblasts in a 1:1 ratio with fresh DMEM-F12+0.5% FCS. After 24h incubation, total RNA was isolated from cardiac fibroblasts using Trizol (Thermo Fisher). For mRNA RT-qPCR, reverse transcription was performed using the High Capacity cDNA Reverse Transcription kit (Thermo, #4368814) and qPCR was performed using Power Up SYBR Green Master Mix (Thermo, # A25779) on a BioRad CFX Connect PCR detection system. Primers for *postn* and *runx2* are listed in Supplemental Table I.

SUPPLEMENTAL TABLE

**Mouse primer**	**Forward**	**Reverse**
Alp3	aacccagacacaagcattcc	gcctttgaggtttttggtca
Aspn	aggacacgttcaagggaatg	aggccttttggaattgaggt
Postn	agtgctctgaggccatcact	aggtcggtgaaagtggtttg
Rplp0	ctctcgctttctggagggtg	acgcgcttgtacccattgat
Runx2	gccgggaatgatgagaacta	ggaccgtccactgtcacttt
Sox9	agctcaccagaccctgagaa	tcccagcaatcgttaccttc

Supplemental Table I. Mouse primer sequences. Alp3: alkaline phosphatase, aspn: asporin, postn: periostin, rplp0: ribosomal protein lateral stalk subunit P0, runx2: runt-related transcription factor 2, sox9: SRY-box transcription factor 9.

	Control 1	Control 2	Control 3	Control 4
**Age**	63	91	67	30
**Sex**	Male	Female	Female	Female
**Diagnosis**	Head & neck cancer	Urinary tract infection, DM	Neurodegenerative disease	Chorioamnionitis, HELLP syndrome
**LVEF %**	60-65%	N/A	N/A	N/A
**Diastolic dysfunction**	N/A	N/A	N/A	N/A
**E/A ratio**	N/A	N/A	N/A	N/A
**Pathology/autopsy: Fibrosis**	N/A	N/A	N/A	N/A
**Pathology/autopsy: Cardiomegaly**	Mild	N/A	N/A	N/A
**Pathology/autopsy: Cardiomyocyte hypertrophy**	N/A	N/A	N/A	N/A
	**HF 1**	**HF 2**	**HF 3**	**HF 4**
**Age**	55	67	62	78
**Sex**	Male	Male	Female	Female
**Diagnosis**	CHF, HTN, ESRD, DM, CAD	CHF, HTN, ESRD, CAD	CHF, DM, CAD	CHF, HTN, DM
**LVEF %**	46	79	N/A	75
**Diastolic dysfunction**	Grade III	Grade II	N/A	Grade I
**E/A ratio**	2.1	1.4	N/A	1.8
**Pathology/autopsy: Fibrosis**	Posterior and anterior LV	Patchy interstitial	Subendocardial, patchy interstitial and perivascular in LV and IVS	Interstitial and perivascular
**Pathology/autopsy: Cardiomegaly**	Yes	N/A	Yes	Yes
**Pathology/autopsy: Cardiomyocyte hypertrophy**	Yes	Yes	N/A	Yes

Supplemental Table II. Patient characteristics. CAD: coronary artery disease; CHF: congestive heart failure; DM: diabetes mellitus; ESRD: end-stage renal disease, HTN: hypertension, LVEF: left ventricular ejection fraction

SUPPLEMENTAL DATA

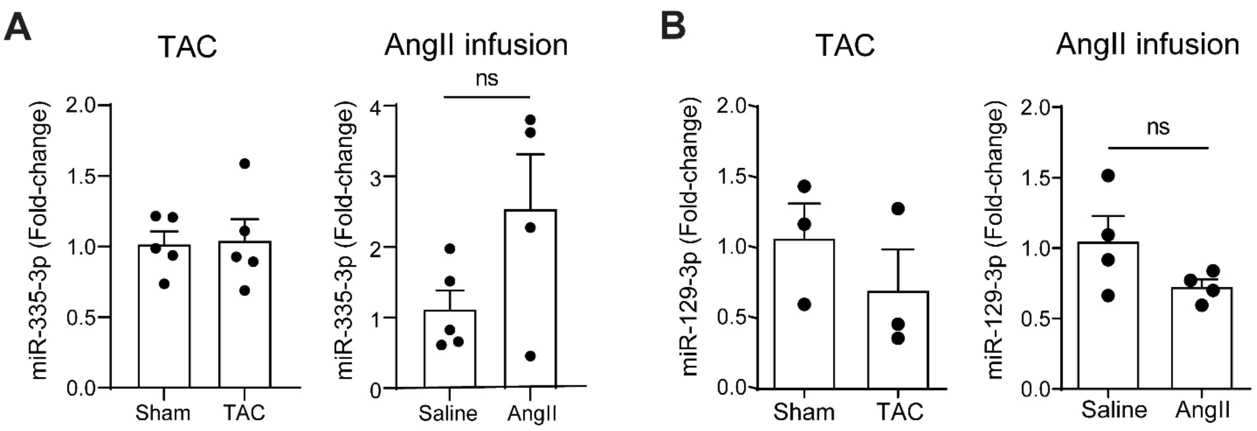

Supplemental Figure I. Validation of bioinformatic analysis. A. miR-335-3p expression in mouse LV measured by qPCR. **B.** miR-129-3p expression in mouse LV measured by qPCR. Data presented as mean+SEM, student's t-test. AngII: Angiotensin II, FACS: flow-assisted cell sorting, Ns: non-significant, TAC: transverse aortic constriction.

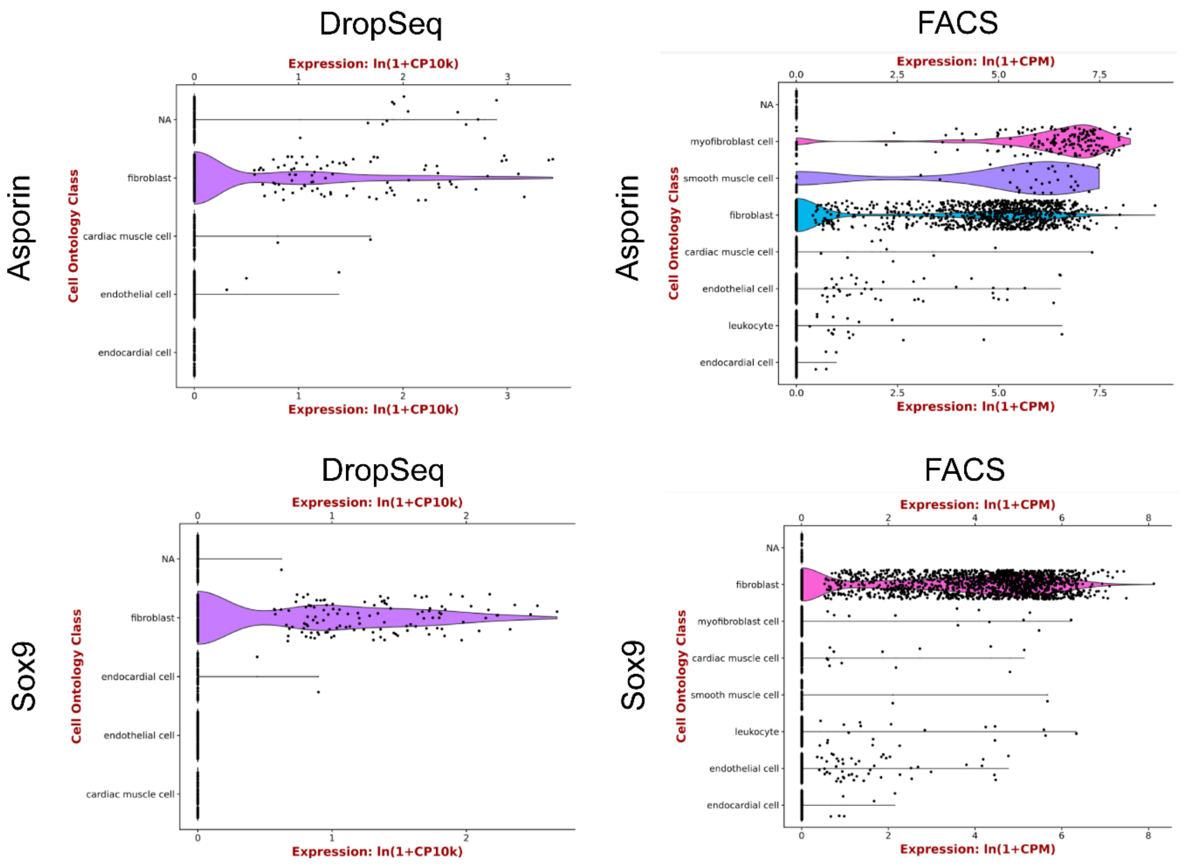

Supplemental Figure II. Expression of Asporin and Sox9 in different cell types of the healthy mouse heart. Measured by DropSeq and FACS in the Tabula Muris publically-available dataset.

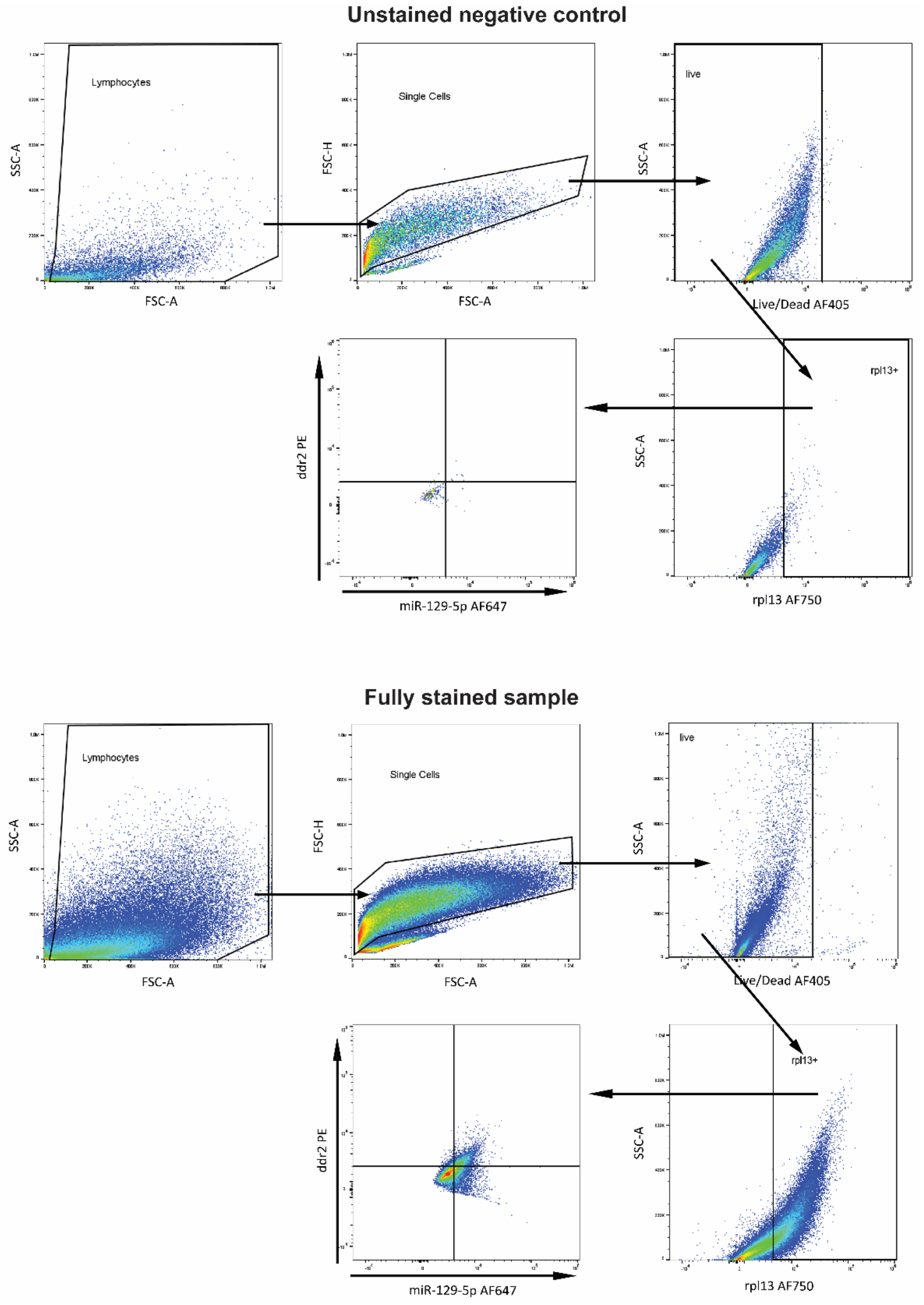

Supplemental Figure III. Gating strategy for PrimeFlow experiments in mouse cardiac single cell suspension with examples of a negative control unstained sample and a fully stained sample.

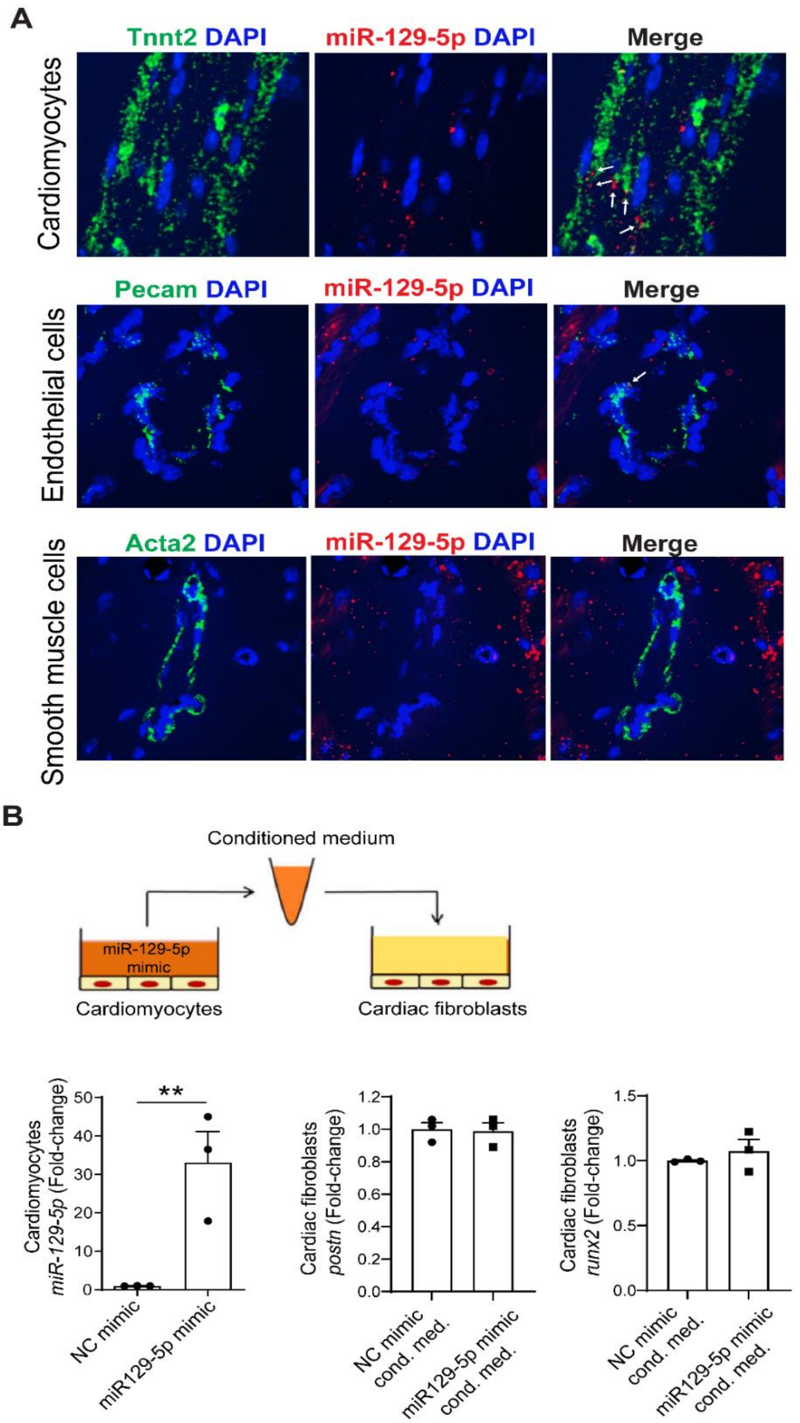

Supplemental Figure IV. miR-129-5p expression in cardiac cell types. A. miR-129-5p expression in cardiomyocytes, endothelial cells, and smooth muscle cells in the healthy mouse hearts as assessed by fluorescence *in situ* hybridization. Tnnt2: cardiac muscle troponin t, Pecam1: platelet and endothelial cell adhesion molecule 1, Acta2: actin alpha 2 smooth muscle **B.** Expression of myofibroblast gene *postn* and osteogenic fibroblast gene *runx2* in cardiac fibroblasts after incubation with conditioned medium from cardiomyocytes differentially expressing miR-129-5p. **>p=0.01, Student's t-tes**t**

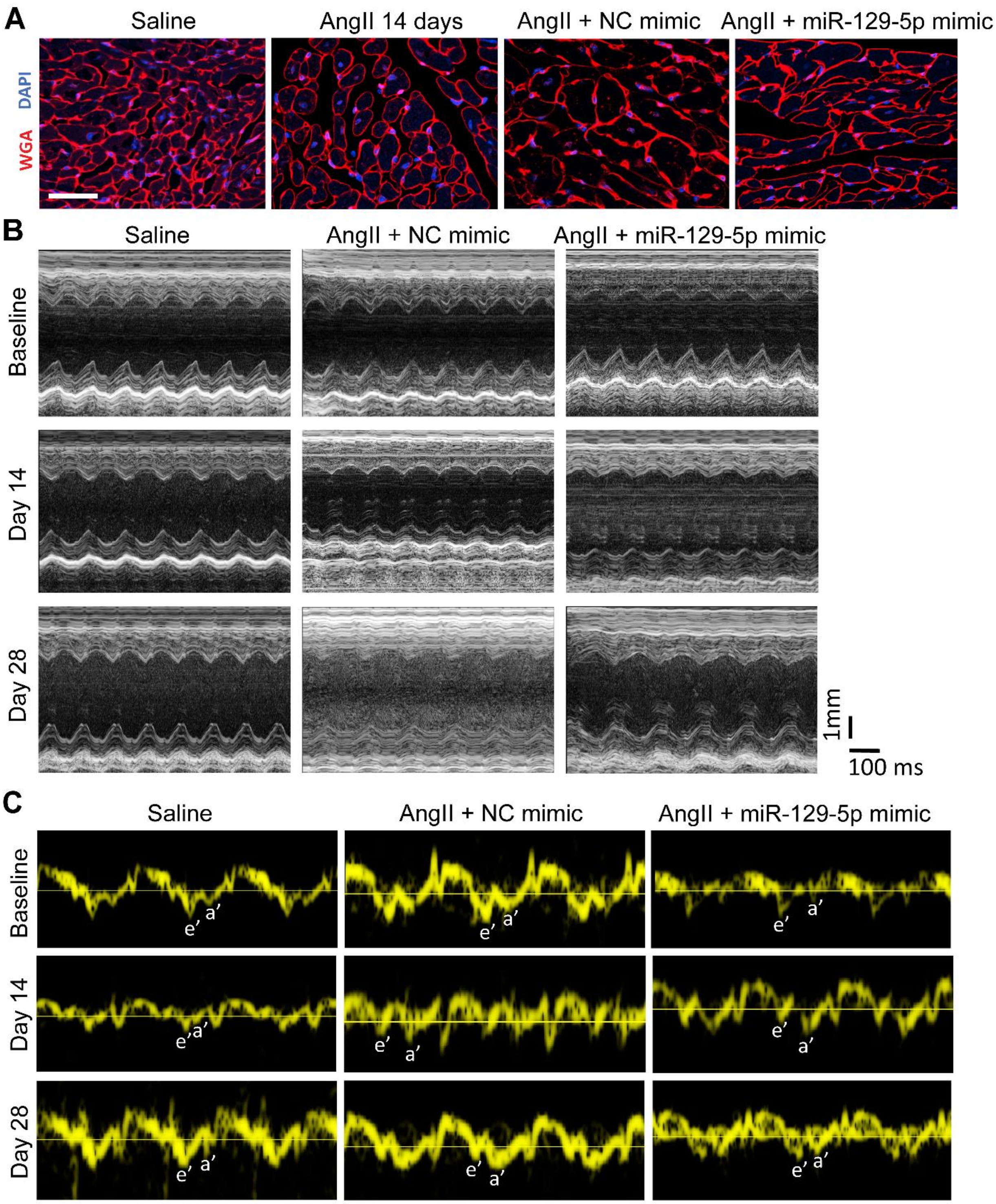

Supplemental Figure V. Cardiac parameters in mice. A. Representative images of cardiomyocyte hypertrophy in mouse hearts assessed by wheat germ agglutinin staining. **B.** Representative M-mode traces. **C.** Representative tissue Doppler traces. **D.** Il-6 expression in hearts of AngII-infused mice treated with negative control or miR-129-5p mimic as assessed by qPCR. *>p=0.05, **>p=0.01, ANOVA with Holm Bonferonni correction. AngII: Angiotensin II. NC: negative contro

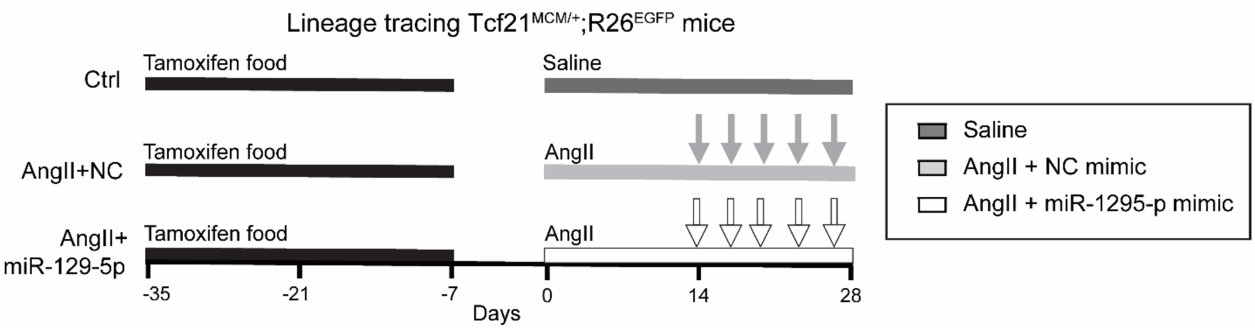

Supplemental Figure VI. Experimental setup for tracing of CF of the transcription factor 21 (Tcf21) lineage in mice ($Tcf21^{MCM/+}$;$R26^{EGFP}$ mice). $Tcf21^{MCM/+}$;$R26^{EGFP}$. Mice were fed a Tamoxifen diet 4 weeks prior to AngII pump implantation to activate eGFP expression in Tcf21-lineage CF. After 14 days of AngII infusion, mice received either miR-129-5p mimics or negative control mimics intravenously every 3 days for the remainder of the experiment.
